# Fruit–frugivore dependencies are important in *Ebolavirus* outbreaks in Sub-Saharan Africa

**DOI:** 10.1111/ecog.06950

**Published:** 2024-04-18

**Authors:** Mekala Sundaram, Mireya Dorado, Benedicta Akaribo, Antoine Filion, Barbara A. Han, Nicole L. Gottdenker, John P. Schmidt, John M. Drake, Patrick R. Stephens

**Affiliations:** 1 Department of Integrative Biology, Oklahoma State University, Stillwater, OK, USA; 2 Department of Infectious Diseases and Savannah River Ecology Laboratory, University of Georgia, Aiken, SC, USA; 3 Northeastern University, Boston, MA, USA; 4 Cary Institute, Milbrook, NY, USA; 5 College of Veterinary Medicine, University of Georgia, Athens, GA, USA; 6 Odum School of Ecology, University of Georgia, Athens, GA, USA; 7 Center for the Ecology of Infectious Diseases, University of Georgia, Athens, GA, USA

**Keywords:** bats, *Ebola*, *Ficus*, frugivore, fruit resources, outbreak, primates, rainfall

## Abstract

Ebolaviruses have the ability to infect a wide variety of species, with many African mammals potentially serving either as primary reservoirs or secondary amplifying hosts. Previous work has shown that frugivorous bats and primates are often associated with spillover and outbreaks. Yet the role that patterns of biodiversity, either of mammalian hosts or of common fruiting species such as *Ficus* (figs, fruit resources used by a wide variety of species), play in driving outbreak risk remains unclear. We investigated what factors most directly influence *Ebolavirus* outbreak risk in Sub-Saharan Africa by using a phylogenetically informed path analysis to compare a wide array of potential models (path diagrams) of spatial dynamics. We considered mammalian frugivore richness, cercopithecid and hominid primate richness, richness of pteropodid (fruit) bats, the spatial distribution of species that have tested positive for *Ebolavirus* antibodies in the wild, *Ficus* habitat suitability, and environmental conditions (mean annual and variability in temperature and rainfall). The proximate factors that most influenced whether a given host species range contained a site of a previous outbreak event were 1) habitat suitability for *Ficus* and 2) the diversity of cercopithecid primates. Frugivore richness overall (including bats, primates, and a few other mammals) and the richness of bats in the family Pteropodidae had a strong effect on which species tested positive for *Ebolavirus* antibodies, but did not influence outbreak risk directly in pathways explored. We interpret this as evidence that foraging around *Ficus* and frugivorous mammals (such as cercopithecid primates which are commonly hunted for food) play a prominent role in driving outbreaks into human communities, relative to other factors we considered which influence outbreak risk more indirectly.

## Introduction

Zoonotic pathogens are transmitted to humans from ‘spillover’ and ‘outbreak’ events that occur due to animal–human contact (Plowright et al. 2017). The majority of emerging human infectious diseases are zoonotic in origin, and the frequency of these events is increasing over time even after accounting for reporting biases (Jones et al. 2008). Zoonotic outbreak events can pose a significant global threat when they evolve into epidemics or pandemics (Morens et al. 2004, Morens and Fauci 2020). Among the most dramatic recent examples was the 2013–2016 West African Ebola epidemic which led to an estimated 28 652 cases and more than 11 000 deaths (CDC 2019). For pathogens such as ebolaviruses that have the potential for such high mortality, it is critical to better understand which factors contribute to outbreak risk.

Ecological mechanisms underlying geographic variation in *Ebolavirus* outbreak risk are relatively unclear. One challenge for ebolaviruses is the absence of a single established reservoir species (Amman et al. 2017). Numerous field surveys have attempted to document infection from exposure to *Ebolavirus* for mammals across Africa (Breman et al. 1999, Leirs et al. 1999, Reiter et al. 1999). However, most species sampled show low, if any, seroprevalence, and *Ebolavirus* infections have been documented in relatively few species (Breman et al. 1999, Leirs et al. 1999, Reiter et al. 1999, Amman et al. 2017, Goldstein et al. 2018, Sundaram et al. 2022). This has led to the conclusion that *Ebolavirus* may be maintained by a network of maintenance hosts rather than a single primary reservoir (Amman et al. 2017). Another challenge is the relative low frequency of documented outbreaks. Armed conflicts and lack of monitoring resources in Sub-Saharan Africa have possibly impeded detection of *Ebolavirus* outbreak events (Stephens et al. 2022, Sundaram et al. 2023). Several additional speculative cases also have been identified where patients in hemorrhagic fever outbreaks showed symptoms consistent with *Ebolavirus* infections, but no lab testing was performed (Kuhn 2008). A handful of studies have attempted to identify ecological mechanisms underlying outbreak risk from ebolaviruses (Olivero et al. 2017, Schmidt et al. 2017, Lee-Cruz et al. 2021). However, there is little consensus, and the influence of some factors such as spatial patterns of host biodiversity have rarely been directly investigated.

**Figure 1.**
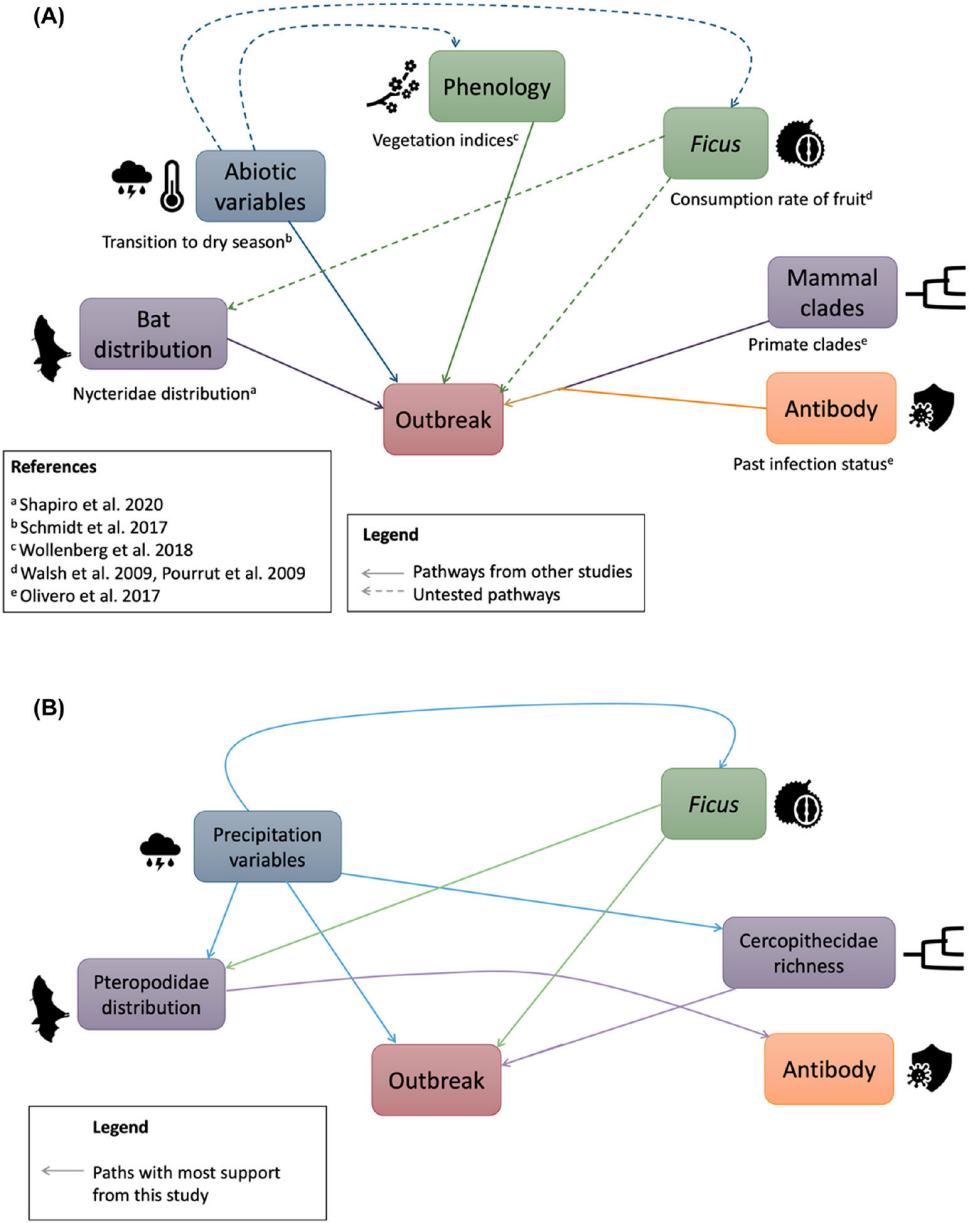
Hypothesized relationships between environmental variables, richness of mammal groups, past infection status of mammals, and fruit resources such as *Ficus* availability on *Ebolavirus* outbreak (A). Relationships with most support provided from this study (B). Colored arrows reflect different types of variables: blue for environment, green for tree/phenology, purple for mammal, and orange for antibody presence.

Several studies noted that transition from rainy to dry seasons appears to be a trigger for outbreak events; possibly because this climatic shift is important for the elusive *Ebolavirus* reservoir (Pinzon et al. 2004, Schmidt et al. 2017) or because transmission of the virus is high under environmental conditions such as high relative humidity and low temperature (Ng and Cowling 2014) (Fig. 1A). However, proposed mechanisms of environmentally linked outbreaks have generally been explored entirely separately from proposed host biodiversity and plant phenology covariates. Models correlating host species occurrences and *Ebola* outbreak events suggest that a range of potential hosts (Fig. 1A) may contribute to outbreaks including African bats, particularly insectivorous bats in the family Nycteridae, and primates (Olivero et al. 2017, Shapiro et al. 2020). While several African bat and primate species have tested positive for *Ebolavirus* infection in the wild (reviewed by Sundaram et al. 2022), no seropositive nycterids have yet been detected nor have any members of this family been associated with primary or index cases from prior outbreaks (Kuhn 2008, Schmidt et al. 2019, Sundaram et al. 2022). Instead, several frugivorous mammals have tested positive for infection (Schmidt et al. 2019, Sundaram et al. 2022), which suggests that animals gathering around fruit resources drive infection (Kuhn 2008). Supporting this theory is the observation that frugivores tend to track fruiting resources across landscapes (Carlo et al. 2013, Fahr et al. 2015). Further, vegetation and phenological changes quantified using remotely sensed vegetation indices have also been linked to increased *Ebolavirus* outbreak risk (Lash et al. 2008, Wollenberg Valero et al. 2018) (Fig. 1A). These studies suggest that changes in plant and tree phenology may trigger outbreaks (Lash et al. 2008, Wollenberg Valero et al. 2018). Although several studies have anecdotally found that frugivory and fruit-producing trees are important in driving *Ebolavirus* outbreaks (Kuhn 2008, Pourrut et al. 2009, Walsh et al. 2009, Schmidt et al. 2019), these hypotheses have remained untested thus far (Fig. 1A). We compared all of these competing variables – environmental predictors, mammalian host richness, and fruiting tree distributions – in conjunction with one another to explore their relative contribution to African *Ebolavirus* outbreak risk.

Using data on both human and epizootic outbreaks, we considered ecological and environmental factors driving outbreak in pathogenic ebolaviruses, by which we refer to African species including *Zaire ebolavirus, Sudan ebolavirus, Tai Forest ebolavirus, Bombali ebolavirus* and *Bundibugyo ebolavirus*, but not *Reston ebolavirus* which is endemic to a different region (Miranda et al. 1999). We combined mammalian host geographic-range information with climatic and *Ficus* tree habitat suitability data to investigate the major drivers of *Ebolavirus* outbreak risk across space. In previous work, Sundaram et al. (2022) evaluated the probable reservoir status of all African mammals using a statistical model based on host traits and phylogenetic relationships. We found that frugivorous species from the families Cercopithecidae (Old World monkeys) and Pteropodidae (fruit bats) were both susceptible and likely to be exposed to ebolaviruses in the wild. In this study, we explored the ecological significance of this finding. *Ficus* fruits are an important component of the diets of species in both groups, and it has been speculated that *Ficus* plays an important role in *Ebolavirus* spread (Formenty et al. 1999, Shanahan et al. 2001, Walsh et al. 2009, 2014, Kagoro-Rugunda and Hashimoto 2015). Using a phylogenetically controlled path analysis, we examined how *Ebolavirus* outbreaks and infection status relate to spatial patterns in the diversity of different potential mammalian reservoir groups, climate, and *Ficus* habitat suitability (Fig. 1A). By comparing the fit of a variety of models (i.e. path diagrams) summarizing potential relationships among these variables, we investigated which factors appear to influence outbreak risk most directly.

## Material and methods

### Response variables and covariates

Zoonotic outbreak events were collected from several sources. Outbreaks up to 2007 were obtained from Kuhn (2008). Subsequent outbreak events into human populations were gathered from CDC (www.cdc.gov/vhf/ebola/history/chronology.html) and georeferenced based on their reported starting locations. Latitude and longitude coordinates for human outbreaks were collected from published literature sources (Mylne et al. 2014, Schmidt et al. 2017, Stephens et al. 2022). To these data we added epizootic outbreaks and their georeferenced locations from Schmidt et al. (2017). The final dataset included n = 37 outbreak events with distinct coordinates. These data are provided on figshare.

We first determined which African mammal ranges overlap zoonotic outbreak events. Mammal geographic ranges were downloaded from the Red List of the International Union for Conservation of Nature (IUCN) (www.iucnredlist.org). A 50 × 50 km grid layer was created for Africa and joined to mammal ranges using the *Spatial Join* function in ArcGIS 10.4 to assess the presence or absence of species in each grid cell. Across the range of each mammal species, we estimated the mean species richness of the mammal groups Pteropodidae, Nycteridae, Bovidae, Cercopithecidae, and Hominidae, and of frugivorous mammals in general (defined as species with > 20% of diet including fruit in EltonTraits, Wilman et al. 2014). For each mammal species, we used ‘Spatial Join’ to determine whether 50 × 50 km grid cells encompassing the entire species range contained at least one *Ebolavirus* zoonotic outbreak event. We assigned each species a value of 1 if its range overlapped an outbreak location, and 0 otherwise. This was our outbreak variable, and a response variable in nearly all models.

Past infection status for African mammals determined from positive antibody and PCR tests was obtained from previous work (Sundaram et al. 2022). We created a past infection status variable of 1 if the species has ever tested positive for *Ebolavirus* and 0 otherwise (see Supporting information for a list of species included). We downloaded global rasters of bioclimatic variables from worldclim (www.worldclim.org), focusing on four that capture central tendencies and variabilities of temperature and rainfall: mean annual temperature (bc1), temperature seasonality (bc4), mean annual precipitation (bc12), and precipitation seasonality (bc15) (Fick and Hijmans 2017). For each mammal range, we calculated the average of each bioclimatic variable. The total number of mammal species with infection status data, geographic ranges, bioclimatic variables, and other covariates in Africa was 210 (see Supporting information for summaries of these mammal species). We then estimated median *Ficus* habitat suitability from georeferenced occurrence points downloaded from the Global Biodiversity Information Facility (GBIF; www.gbif.org) and average *Ficus* fruit volume in each mammal range from published fruit measurements of African figs (Berg and Wiebes 1992). We chose median for habitat suitability due to an extreme skew in spatial variation of this layer. However, we ensured that our final results were robust to the use of median measurements for all variables explored. Our process to estimate *Ficus* habitat suitability is described in the next section.

We created global and local African *Ficus* habitat suitability maps from occurrence points using distribution models. We downloaded *Ficus* latitude and longitude points from GBIF. All *Ficus* coordinates used for analyses are available on figshare. We removed rows where the basis of record was ‘observation’ (rather than a specimen identified by a professional taxonomist) or ‘fossil specimen’ (since we were interested in the current range). We filtered final points to rows where *Ficus* presence points were georeferenced to latitude and longitude and used these data to create habitat suitability maps with distribution models described in the next section.

First, we fit distribution models to all *Ficus* points across the globe. This global model with Area under the curve (AUC) = 0.86, and the resulting habitat suitability map was clipped to Africa for further analyses. Our second model was fit only to *Ficus* points from Africa with AUC = 0.87. For each dataset, we estimated habitat suitability using ensemble distribution modelling methods, which is a weighted average of suitabilities computed from different modelling algorithms (more details are provided in the next paragraph). To test the robustness of our results to estimated *Ficus* habitat suitability layers, further statistical analyses of drivers of Ebola outbreaks were repeated using habitat suitability maps estimated both globally and using only African occurrences. We also repeated our analyses with richness of *Ficus* species estimated by georeferencing and digitizing ranges of African species to a resolution of ~ 20 km from geographic range maps published on figweb.org (van Noort and Rasplus 2022). The resulting shapefiles are also available on figshare.

Ensemble distribution models of *Ficus* were fit using environmental rasters of mean annual temperature, annual precipitation, and precipitation seasonality as predictors. We dropped temperature variability from the final models due to a high degree of correlation with mean annual temperature (Pearson’s R > 0.75). Final ensemble distribution models were fit with two algorithms, maximum entropy (MAXENT) and multivariate adaptive regression splines (MARS) in R package ‘SSDM’ (Schmitt et al. 2017, 2020). We also considered gradient boosted model algorithms, but discarded these models due to poor predictive accuracy (i.e. AUC < 0.7) compared to MAXENT and MARS. Following recommendations from previous studies, we chose 10 000 random points as pseudoabsences (Barbet-Massin et al. 2012, Schmitt et al. 2017). For all models, we assumed equal weighting of presences and absences. We evaluated fit as the mean accuracy across all models and using 1000-fold cross validation and 1000 repeats (Schmitt et al. 2017). Hyper-parameters for algorithm fitting were based on recommendations of studies (Friedman 1991, Phillips et al. 2006, Elith et al. 2011, Barbet-Massin et al. 2012, Schmitt et al. 2017, Milborrow 2020), including convergence threshold of 0.00001, 1000 maximum iterations, and a regularization beta value of 0.0001 for MAXENT. We also allowed the algorithm to choose any feature construction, i.e. product, binary, quadratic, and linear (Phillips et al. 2006, Elith et al. 2011). For MARS models, we set maximum degree of interaction between variables to two (Schmitt et al. 2020), we chose the default forward stepping threshold 0.001, and we computed maximum number of terms and number of observations allowed between knots from default formulae based on number of predictors and total observations (Friedman 1991, Milborrow 2020). Pruning was performed with backward elimination which estimates fit using cross validation (Golub et al. 1979, Milborrow 2020). We did not remove duplicate points that fall in the same grid cell of raster. Final *Ficus* habitat suitability maps are shown in the Supporting information. Estimated spatial patterns of *Ficus* habitat suitability also closely matched patterns of *Ficus* species richness based on species distributions reported in figweb.org (van Noort and Rasplus 2022).

### Overview of phylogenetic path analysis

To investigate relationships between mammal richness variables, environmental variables, *Ficus* habitat suitability, past infection status, and *Ebolavirus* outbreak occurrences, we performed a phylogenetic path analysis (van der Bijl 2018). This method allowed paths of varying complexity to be compared using a metric similar to the Akaike information criterion (AIC) (below), while also accounting for the potential statistical influence of host phylogenetic relationships. This regression approach accommodates both continuous and binary variables (Ho and Ané 2014, van der Bijl 2018). The phylogeny used for analyses was the maximum clade credibility tree of a recent comprehensive analysis of all mammals (Upham et al. 2019). Phylogenetic signal was computed for the continuous variables using Pagel’s λ (Pagel 1999) and with a transition matrix-based estimate of phylogenetic correlation for binary variables (Ives and Garland 2010). Both were implemented in a regression framework (Ho and Ané 2014).

We constructed a series of path diagrams of increasing complexity to explore the factors explaining which species have tested positive for *Ebolavirus* infection and which species ranges overlap known zoonotic outbreak events. These analyses were performed at a species level (i.e. each row of data in our model was a species) predicting infection status and outbreaks (both binary variables) as a function of continuous covariates averaged (or median computed) across the species range. We first conducted analyses with only biological variables in order to explore how the richness of different groups of mammals and *Ficus* habitat suitability predicted infection status and geographic overlap with sites of outbreaks (see ‘Pathways with biological variables’ for details about specific pathways and hypotheses tested). We then conducted analyses with both biological and environmental variables to explore how both suites of variables influenced infection status and outbreak occurrence (more details in ‘Pathways with biological and environmental variables’). We constructed all acyclic path diagrams and tested for significance of path coefficients in a phylogenetic comparative framework using R package ‘phylopath’ (van der Bijl 2018).

### Pathways with biological variables

We first conducted analyses including only biological variables. The response variable was binary where 1 = outbreak site occurs within a species range and 0 = outbreak site does not occur in a species range. Our predictor variables were pteropodid richness, cercopithecid richness, bovid richness, nycterid richness, *Ficus* habitat suitability, *Ficus* fruit volume, and frugivore richness within each species range, as well as infection status determined from antibody and PCR tests (1 = infected, 0 = infection absent). We refer to the latter using ‘antibody’ in Fig. 1 because this is the method most frequently used to test infection status. Our hypothesized pathways were based on relationships shown by previous workers and summarized in Fig. 1A. Given that previous studies identified Nycteridae, Bovidae, and species testing positive for infection as important predictors of outbreak risk (Fig. 1A), we incorporated richness of these groups as potential drivers of whether species ranges overlapped with outbreak sites in hypothesized pathways (Supporting information). We also included *Ficus* habitat suitability and fruit volumes in these pathways to explore whether higher potential fruit abundance and presence of larger fruit drive outbreak risk (Supporting information).

Once we had examined the influence of broader patterns of host and *Ficus* biodiversity, we tested for a direct effect of past infection on outbreak risk. We examined whether species that had been shown to have been infected with Ebolaviruses in the past using antibodies or PCR were more likely to contain sites of outbreaks within their ranges. We compared the fit of models in which infection status (0 or 1, and noted as ‘ab’ in path diagrams since antibodies have been used for testing most frequently) directly influences outbreak occurrence to models in which it does not. In the latter models infection status essentially occurs in the model as an additional response variable. A direct effect would imply that the presence of species shown to have been exposed and susceptible to ebolaviruses in the past can predict where the opportunity for outbreaks (i.e. exposure of human populations to the virus) is high somewhat independently of broader overall patterns of biodiversity. See Supporting information for all 21 pathways of biological variables tested.

### Pathways with biological and environmental variables

We next included environmental variables (bc1: mean annual temperature, bc12: annual precipitation, bc4: temperature seasonality, bc15: precipitation seasonality) in a second series of path diagrams to examine if environmental variables directly predicted outbreak risk, as has been suggested previously (Fig. 1A), or if environmental variables only influence outbreaks indirectly by influencing spatial patterns of biodiversity. The Supporting information shows all pathways examining direct effects versus indirect effects of environmental variables on outbreak risk with *Ficus* layers predicted from global points. We also repeated analyses of pathways both including and excluding environmental variables using local *Ficus* habitat suitability based only on georeferenced points in Africa (as opposed to all *Ficus* globally), to test sensitivity of results to the method used for estimation of *Ficus* habitat suitability. The Supporting information shows pathways testing direct versus indirect effects of environmental variables where *Ficus* layers were estimated from Africa points only. We only report the overall best models in the main text; see the Supporting information for all paths considered using both sets of *Ficus* distribution models. Finally, we repeated analyses with *Ficus* richness georeferenced from figweb.org, which yielded qualitatively similar results to analyses based on estimated *Ficus* suitability (see Supporting information for more details).

We evaluated pathways based on their C-statistic information criteria or CICc scores, which is a Fisher’s C-statistic corrected for number of parameters included in pathway (van der Bijl 2018). Like AIC, models with lower scores are considered better supported, and models separated by fewer than four points (i.e. ΔCICc < 4) are considered to have equivalent support (von Hardenberg and Gonzalez-Voyer 2013, Gonzalez-Voyer and von Hardenberg 2014, van der Bijl 2018). Final selected path diagrams also had to pass the d-separation test, which tests whether pathways that are missing in a given diagram influence the response variable independently of variables already present in a pathway (Gonzalez-Voyer and von Hardenberg 2014, van der Bijl 2018). We also used machine learning to evaluate how well variables in the final pathways performed in predicting outbreak occurrence in a mammal range (1 = outbreak occurs in range, 0 = outbreak absent in range). We performed ridge regressions with 10-fold cross validation repeated 100 times, implemented using the R package ‘caret’ (Kuhn et al. 2020). We report the AUC values of the resulting models as a measure of which path diagrams and variables best predict outbreak risk. We enforced a balanced sampling strategy during these analyses, with presence localities compared to an equal number of random pseudoabsence localities in each fold and replicated to create an ensemble of models to avoid inflating observed AUC scores.

## Results

### Pathways with biological variables

**Figure 2.**
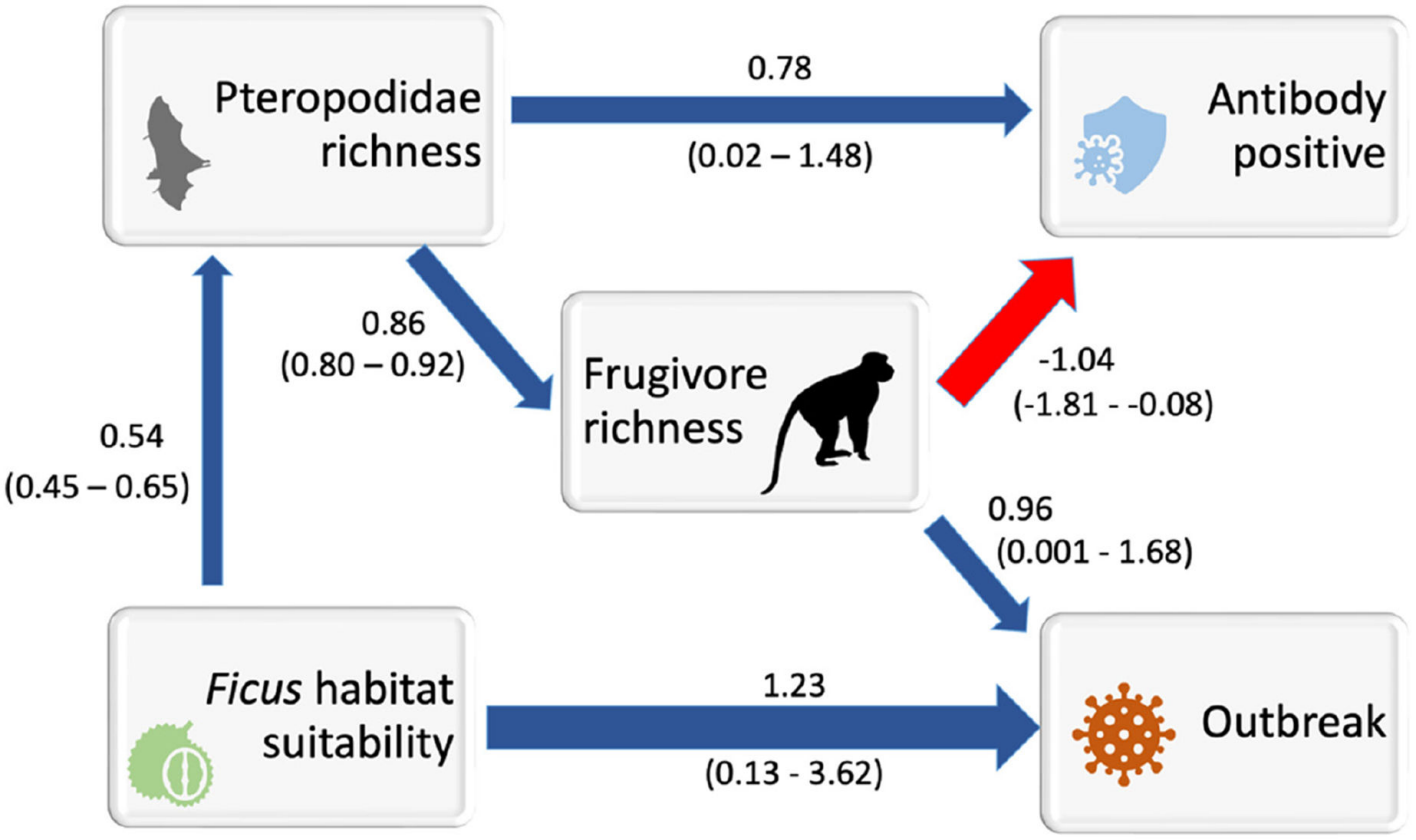
Simplest path diagram depicting relationships between all biological variables (*Ficus* habitat suitability modelled using global distribution of all trees, frugivore richness, Pteropodidae richness, past infection status of mammal to *Ebolavirus*), and overlap of range with *Ebolavirus* outbreak site. Path coefficients are presented only for those links that are significantly different from 0 (see Supporting information for details). Red arrow shows negative path coefficient and blue shows positive path coefficient. This pathway is the best supported pathway from Table 1 with lowest CICc value.

*Ficus* habitat suitability computed from the global distribution of georeferenced points was positively and directly related to *Ebolavirus* outbreak occurrence in mammal ranges when biological variables were tested (Table 1, model seven had lowest CICc value). We first tested a wide range of biodiversity drivers in order to narrow down which specific mammal and biological drivers were most important in explaining outbreaks (see Supporting information for pictorial representation of all pathways). The model with the lowest CICc value included Pteropodid richness, *Ficus* habitat suitability, frugivore richness, infection status determined from antibody and PCR tests, and outbreaks occurring in mammal ranges (Supporting information), whereas richness of Nycteridae and Bovidae were excluded. Average *Ficus* fruit volume was also excluded at this stage (Supporting information). Next, we tested a series of pathways to examine if positive infection directly influenced outbreak occurrence where the best pathway was selected based on CIC values and d-separation tests (see Supporting information for full pathways tested). This pathway suggested that outbreak occurrence within African mammal ranges was positively and directly related to average frugivore richness and *Ficus* habitat suitability (path-7 in Table 1, Fig. 2; bootstrap intervals around path coefficients provided in Fig. 2 when the relationship is significantly different from 0). In contrast, the ranges of mammals that were antibody positive occurred in areas of high average pteropodid richness or regions where fruit bat richness was highest (Fig. 2). This pathway (Fig. 2) passed d-separation tests (Table 1 D sep p = 0.53; see Supporting information for detailed breakdown of d-separation tests) and had the highest model weight (w = 1.000), lowest CICc score (CICc = 31), and showed a ΔCICc of 138 compared to the next best model (Table 1). Frugivore richness and *Ficus* habitat suitability predicted outbreak occurrence with an AUC of 0.78 in ridge regression models.

### Pathways with biological and environmental variables

**Figure 3.**
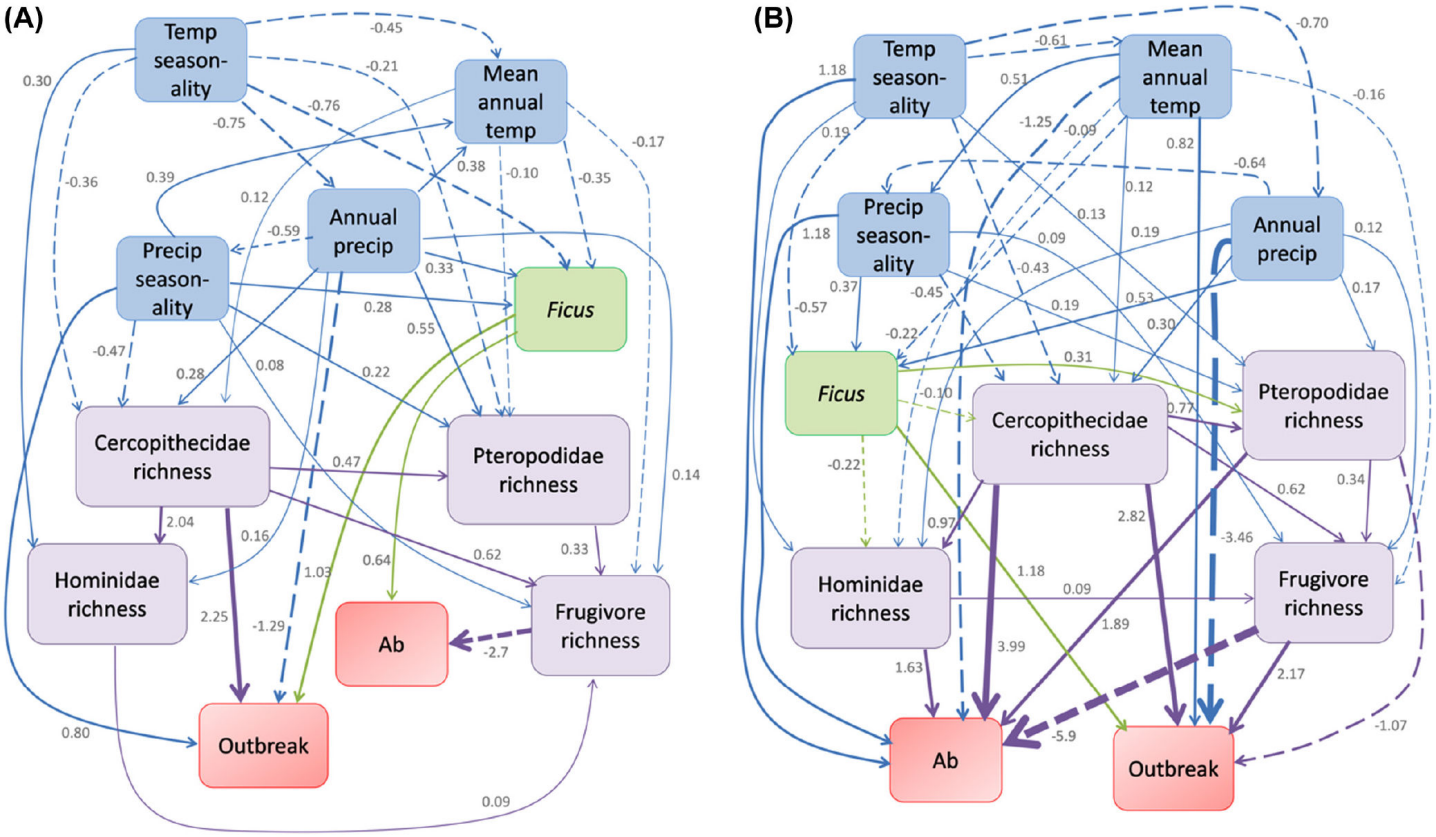
Path diagram depicting relationships between *Ficus* habitat suitability, richness, of different mammal clades, environmental variables, past infection status of mammal to *Ebolavirus* (shown as ‘Ab’), and overlap of range with *Ebolavirus* outbreak site (shown as ‘Outbreak’). Path coefficients are presented only for those links that are significantly different from 0. Solid lines are paths with significant positive coefficients and dotted lines are paths with significant negative coefficients. (A) Final path diagram using *Ficus* suitability modelled with global distribution of all *Ficus* species (corresponds to best pathway in Table 2); and (B) final path diagram using *Ficus* suitability modelled with distribution of *Ficus* from Africa only (corresponds to best pathway in Table 3). Colored arrows reflect different types of variables: blue for environment, green for tree distributions, purple for mammal, and red for response variables of outbreak occurrence and antibody presence.

The addition of environmental variables to mammal biodiversity, and Ficus suitability, increased the complexity of the pathways; however, Ficus habitat suitability and richness of multiple frugivorous mammals still predicted outbreak occurrence (Fig. 3A; see Supporting information for bootstrap confidence intervals around path coefficients). We examined multiple pathways including all variables (Supporting information). The best pathway (path 13) predicted that both outbreak and past infection were associated with Ficus habitat suitability, richness of mammals, and environmental variables (Table 2). Mammal ranges containing zoonotic outbreak events overlapped regions of high Ficus habitat suitability and high cercopithecid richness (Fig. 3A; see Supporting information for bootstrap intervals around path coefficients). Mammal ranges in regions of low annual precipitation and high seasonality in precipitation were also related to outbreak occurrences (Fig. 3A). Whether species have tested positive for Ebolavirus infections using either PCR or antibody tests, the latter reflecting either current or previous infections, did not directly influence whether species ranges overlapped areas of known outbreak events (Fig. 3A). However, high Ficus habitat suitability within ranges did explain which species have tested positive for infection (Fig. 3A). Even though the directionality of relationships between environmental variables are difficult to ascertain, switching directions in tested pathways altered path coefficients minimally. Our final best path in Fig. 3A was path 13, which had the most support with model weight = 0.44 and lowest CICc = 190 (Table 2). This path also passed d-separation tests (Overall d-sep p = 0.09, see Supporting information for detailed breakdown of d-separation tests), suggesting that no relationships were missing from our final path diagram. In ridge regression models, the precipitation, seasonality in precipitation, Ficus habitat suitability, and Cercopithecidae richness together predicted outbreak occurrence with AUC = 0.796.

Results of analyses using *Ficus* richness determined from digitizing ranges on figweb.org provided qualitatively similar results to the models using global *Ficus* habitat suitability (Supporting information). The same path (seven, see Supporting information) among simpler models that included only biological variables was most strongly supported and path thirteen from among the more complex paths that included both biological and environmental variables was supported (Supporting information). The AUC scores from ridge regressions were also similar, with an AUC score of 0.79 when environmental variables were excluded, and 0.83 when they were included.

*Ficus* habitat suitability estimated from a model with only African species still predicted outbreak occurrence (Fig. 3B). After rejecting numerous alternative paths (Supporting information), our final model (model-11, Table 3) found both high *Ficus* habitat suitability and Cercopithecidae richness to be associated with outbreaks (Fig. 3B). This path also suggests that ranges overlapping regions of lower precipitation and high temperature were related to outbreaks (Fig. 3B). Species testing positive for past *Ebolavirus* infection occurred in regions of high cercopithecid, pteropodid, and hominid richness. These species also occurred in areas of high seasonality in precipitation and lower mean annual temperatures (Fig. 3B). Bootstrap intervals around all path coefficients were significantly different from 0 (Supporting information). This model (Fig. 3B) was best supported with a weight of nearly 1.0 (Table 3) and passed the d-separation test (d-sep p = 0.581; see Supporting information for breakdown of d-separation tests). Precipitation, temperature, Cercopithecidae richness, and *Ficus* habitat suitability predicted outbreak occurrences with AUC = 0.80.

## Discussion

The factors driving *Ebolavirus* outbreaks have been challenging to characterize thus far because of the absence of a confirmed primary reservoir. Here, we explore how a range of mammal richness variables, environmental variables, and data about fruiting tree resources predict zoonotic outbreak events of *Ebolavirus.* We find that fruiting resources such as *Ficus* and fruit eating mammals such as monkeys from Cercopithecidae relate to outbreak risk.

Our best supported path diagrams suggest that pteropodid bat richness correlates to infection status, both when biological variables only were considered and when environmental variables were included (Fig. 2-3B). Previous studies have suggested that bats play a role in outbreaks; however, there has been considerable uncertainty in linking bat families to outbreak sites (Olivero et al. 2017, Shapiro et al. 2020). Fruit bats have often been considered the most likely primary *Ebolavirus* reservoirs because some species have tested positive for infection in the wild, and species tested in lab inoculation experiments also tolerate infections well (Leroy et al. 2005, Hayman et al. 2012, Formenty 2014, Hassanin et al. 2016, Olivero et al. 2020, Sundaram et al. 2022). Nevertheless, very few, if any, actual case of outbreaks in human populations have been tracked directly back to fruit bats (Kuhn 2008). Most zoonotic outbreak cases occur from contact with other frugivorous mammals that experience lethal infection, and serve as dead-end hosts, such as primates (Rouquet et al. 2005, Kuhn 2008). Our path analyses support the idea that pteropodid bats do not directly influence outbreaks. Instead, outbreak events occur in mammal ranges where cercopithecid richness or overall frugivore richness is especially high (Fig. 2–3). However, our path analyses also suggest that pteropodid fruit bat richness is high in ranges of species that have tested positive for *Ebolavirus* infections (Fig. 2–3). The more complex path analyses suggest richness of monkeys from Cercopithecidae and richness of apes from Hominidae are also associated with past infections (Fig. 3B) or that availability of fruiting resources such as *Ficus* is associated with infections (Fig. 3A). Overall, our results suggest that fruit resources could play an important role in *Ebolavirus* transmission, particularly from pteropodid bats to other hosts, such as cercopithecids and hominids, and ultimately to humans. A previous comprehensive study of all African mammals also highlighted pteropodids as strong candidates for primary *Ebolavirus* reservoirs based on their ecological and life history characteristics (Sundaram et al. 2022).

Zoonotic outbreak risk of ebolaviruses appears to be most closely linked to *Ficus* habitat suitability and cercopithecid richness. Our path analyses confirm an association between *Ficus* habitat suitability and outbreaks (Fig. 3), but also *Ficus* habitat suitability and infection status from antibody and PCR tests (Fig. 3A). Walsh et al. (2009) previously hypothesized that fruit resources such as *Ficus* play a role in spread of *Ebolavirus*, but follow-up tests of this hypothesis are difficult to undertake in the wild. Several frugivorous mammals from groups Cercopithecidae, Pteropodidae, and Hominidae have tested positive for infection from *Ebolavirus* in the wild (Sundaram et al. 2022). Many of these species have *Ficus* common in their diet because members of the genus *Ficus* fruit year-round and together serve as a consistently available fruit source for mammals in Sub-Saharan Africa (Berg and Wiebes 1992, Formenty et al. 1999, Kirika et al. 2008, Leroy et al. 2009, Walsh et al. 2009). For example, partial sequences of *Ebolavirus* have been isolated from *Hypsignathus monstrosus*, a pteropodid bat species which is known to specialize on fig fruits (Bradbury 1977, Leroy et al. 2005). Further, some fruit bats such as *Epomophorous wahlbergii* have been found to track ripe fig sources across Africa (Bonaccorso et al. 2014). Frugivore movements are considered to be specific and directional in favor of forested areas (Carlo et al. 2013). We suggest that sites with ripe *Ficus* fruit may represent locations where infection spreads to primates (Walsh et al. 2009), ultimately leading to outbreaks in humans (Kuhn 2008). This mechanism of frugivores gathering around figs maybe more pronounced in areas adjacent to specific crop types, as has been shown in other study systems (Deshpande et al. 2022, Eby et al. 2023). Compared to other tropical fruits, *Ficus* fruits are also rich in calcium, which has been shown to increase *Ebolavirus* infectivity at a cellular level (Nathan et al. 2020). In addition to *Ficus*, our path analyses specifically suggest that regions where cercopithecid richness is high are important in predicting outbreaks (Fig. 3). Several cercopithecids have tested positive for past infection from *Ebolavirus* (Ayouba et al. 2019, Sundaram et al. 2022), and future work exploring spatial and temporal overlap with fruit bats during *Ficus* foraging may shed light on how these species typically become infected.

We also examined environmental variables in our path analyses. When performing analyses with different mammal families and *Ficus* habitat suitability, environmental variables influenced outbreak both indirectly through *Ficus* habitat layers and richness of mammal groups, and directly (Fig. 3). Precipitation-related variables, specifically patterns of mean annual precipitation, were found to be consistently negatively associated with outbreak, and ranges that overlap areas of lower rainfall were more likely to overlap outbreak locations (Fig. 3A-B). We interpret this finding to mean that any influence of environment variables on outbreak most likely represents an influence on fruit production, as has been suggested by Wollenberg Valero et al. (2018). Further, we speculate that areas of low rainfall and high seasonality in precipitation may represent those areas where frugivores congregate after having consumed the more perishable and seasonal fruits available in other areas (Vander Wall 1990, Isbell 1991, Utami et al. 1997, Snaith and Chapman 2005). More information pertaining to fruit resources and their distributions in Africa will be important for testing this specific hypothesis.

While previous studies have found Nycterid bats (Shapiro et al. 2020) and environmental variables (Schmidt et al. 2017) to be important predictors of outbreak risk (Fig. 1A), we did not find support for non-frugivorous mammals in *Ebolavirus* outbreaks (Fig. 2-3). Although our results are based on diversity of mammals as opposed to abundance, we predict comparable and perhaps more pronounced results for frugivore abundance and fruit abundance. Certainly, we found support for the same pathways regardless of whether *Ficus* was represented via habitat suitability (Table 1-3) or via *Ficus* richness (Supporting information). Taken together, our results suggest that congregation of fruit eating mammals around fig trees may lead to spread of ebolaviruses to animals. This, in turn, ultimately drives outbreaks in human populations, when individuals go to the same areas to gather fruit or to hunt primates, particularly cercopithecids (Wolfe et al. 2004, N’Goran et al. 2012, Covey and Scott McGraw 2014), for meat. There is some evidence that insectivorous bats also play a role in outbreak of ebolaviruses and test positive for infection (Saéz et al. 2015, Goldstein et al. 2018, Sundaram et al. 2022). Our path analyses suggest that, like primates, these species may have been exposed to Ebola from overlap with pteropodid fruit bats and may thus serve as proximate reservoirs for outbreaks.

In summary, we find support for the hypothesis that Pteropodid fruit bats are likely the primary reservoirs of Ebola, transferring the pathogen to other secondary reservoirs such as insectivorous bats, cercopithecids and hominids (Fig. 1B). We hypothesize that frugivorous mammals spread infection by gathering around fruiting resources such as *Ficus*. As stable and abundant fruit resources, *Ficus* species may be of particular importance during periods of low precipitation and in areas where precipitation is seasonal (Fig. 1B). Ultimately, outbreaks of Ebola into new populations including in humans is driven by richness of secondary hosts such as cercopithecids in areas where *Ficus* occurs (Fig. 1B). While we offer the first comprehensive test of previously hypothesized pathways underlying outbreaks, detailed behavioral data pertaining to frugivorous mammals in Africa such as food handling, fission-fusion foraging strategies, foraging on ‘dropped fruits and seeds’, and anthropogenic changes in fruit availability (Eby et al. 2023) will likely provide important mechanistic insights on how ebolaviruses can spread.

## Supplementary Material

Supplementary material

## Figures and Tables

**Table 1.** Results for the pathways testing biological variables and infection status (tested via antibodies) on outbreak occurrence. For each path, the table summarizes the model number (model) with abbreviated pathway, the number of independence tests performed (k), the number of parameters in the model (q), C-statistic (C), D sep p value associated with d separation tests (p), C-statistic corrected for small sample sizes (CICc), difference between model CICc value and the smallest CICc value in set (ΔCICc), and weight of model relative to other models in set (w). Abbreviations for pathways include ‘OU’ for outbreak, ‘AB’ for past infection status, ‘FIC’ for *Ficus* habitat suitability, ‘FRUG’ for frugivore richness, ‘PTERO’ for Pteropodidae richness, ‘CERCO’ for Cercopithecidae richness, ‘HOM’ for Hominidae richness, ‘ANIMALS’ for all groups of animals (namely Pteropodidae richness, Cercopithicidae richness, Hominidae richness), ‘ENV’ for all environmental variables, ‘MAT’ for mean annual temperature, and ‘P’ for precipitation. See Supporting information for details of full pathways tested corresponding to model numbers presented below, and additional pathways tested but not included in this table. *Ficus* distribution was estimated from global points.

Model		q	C	D sep p	CICc	ΔCICc	w
7: OU ~ AB + FIC + FRUG	2	13	3.16	0.531	31	0	1.00E + 00
2: OU ~ FRUG, AB ~ FRUG	6	9	150.36	0	169	138	< 0.0001
5: OU ~ FIC	6	9	157.63	0	177	146	< 0.0001
6: AB ~ FIC + PTERO	6	9	216.28	0	235	204	< 0.0001
4: OU ~ FRUG + FIC	5	10	219.12	0	240	209	< 0.0001
8: OU ~ FIC + AB	6	9	224.91	0	244	213	< 0.0001
3: AB ~ PTERO + FRUG	8	7	315.38	0	330	299	< 0.0001
1: AB ~ FIC + FRUG + PTERO	7	8	314.62	0	331	300	< 0.0001

**Table 2.** Results for the pathways testing biological variables, environmental conditions, and infection status (tested via antibodies) on outbreak occurrence. *Ficus* habitat suitability was estimated from global points of *Ficus* occurrence. For each path, the table summarizes the model number (model) with abbreviated pathway, the number of independence tests performed (k), the number of parameters in the model (q), C-statistic (C), D sep p value associated with d separation tests (p), C-statistic corrected for small sample sizes (CICc), difference between model CICc value and the smallest CICc value in set (ΔCICc), and weight of model relative to other models in set (w). Abbreviations for pathways include ‘OU’ for outbreak, ‘AB’ for past infection status, ‘FIC’ for *Ficus* habitat suitability, ‘FRUG’ for frugivore richness, ‘PTERO’ for Pteropodidae richness, ‘CERCO’ for Cercopithecidae richness, ‘HOM’ for Hominidae richness, ‘ANIMALS’ for all groups of animals (namely Pteropodidae richness, Cercopithicidae richness, Hominidae richness), ‘ENV’ for all environmental variables, ‘MAT’ for mean annual temperature, and ‘P’ for precipitation. See Supporting information for the full pathways tested corresponding to model numbers presented below, and for additional pathways tested but not included in this table.

Model		q	C	D sep p	CICc	ΔCICc	w
13: OU & AB ~ ENV + FIC + ANIMALS	5	61	16.3	0.0901	190	0	0.439
11: OU & ANIMALS & AB ~ ENV + FIC	6	60	20.6	0.0563	190	0.268	0.384
14: OU ~ AB + ENV + FIC + ANIMALS,	3	63	10.2	0.1151	192	2.044	0.158
12: OU ~ ENV + FIC + ANIMALS	4	62	18.7	0.0166	196	6.388	0.018
10: OU & AB & ANIMALS ~ ENV	18	48	482.8	0	608	418.378	< 0.0001
15: OU ~ FIC + AB, AB & ANIMALS ~ ENV	22	44	505.9	0	618	428.266	< 0.0001
5: OU ~ AB, AB & ANIMALS ~ ENV	22	44	524.2	0	636	446.562	< 0.0001
6: OU ~ AB, AB & ANIMALS ~ ENV + FIC	22	44	591.1	0	703	513.47	< 0.0001
9: OU ~ HOM, AB & ANIMALS ~ ENV + FIC	24	42	692.2	0	798	608.152	< 0.0001
8: OU ~ FIC + FRUG, AB & ANIMALS ~ ENV + FIC	24	42	767.7	0	873	683.609	< 0.0001
16: OU ~ AB + FIC, AB ~ ANIMALS ~ ENV	24	42	772.8	0	879	688.712	< 0.0001
4: OU ~ CERCO, AB & ANIMALS ~ ENV	26	40	783.8	0	883	693.525	< 0.0001
2: OU ~ FRUG, AB & ANIMALS ~ ENV	25	41	788	0	891	700.861	< 0.0001
3: OU ~ PTERO + FRUG + CERCO, AB & ANIMALS ~ ENV	24	42	785.4	0	891	701.352	< 0.0001
1: OU ~ PTERO, AB & ANIMALS ~ ENV	26	40	796.8	0	896	706.542	< 0.0001
7: OU ~ FIC, AB ~ ANIMALS ~ ENV + FIC	25	41	793.9	0	897	706.75	< 0.0001

**Table 3.** Results for the pathways testing biological variables, environmental conditions, and infection status (tested via antibodies) on outbreak occurrence. *Ficus* habitat suitability was estimated from points of *Ficus* occurrence in Africa only. For each path, the table summarizes the model number (model) with abbreviated pathway, the number of independence tests performed (k), the number of parameters in the model (q), C-statistic (C), D sep p-value associated with d separation tests (p), C-statistic corrected for small sample sizes (CICc), difference between model CICc value and the smallest CICc value in set (ΔCICc), and weight of model relative to other models in set (w). Abbreviations for pathways include ‘OU’ for outbreak, ‘AB’ for past infection status, ‘FIC’ for *Ficus* habitat suitability, ‘FRUG’ for frugivore richness, ‘PTERO’ for Pteropodidae richness, ‘CERCO’ for Cercopithecidae richness, ‘HOM’ for Hominidae richness, ‘ANIMALS’ for all groups of animals (namely Pteropodidae richness, Cercopithicidae richness, Hominidae richness), ‘ENV’ for all environmental variables, ‘MAT’ for mean annual temperature, and ‘P’ for precipitation. See Supporting information for the full pathways tested corresponding to model numbers presented below, and for additional pathways tested but not included in this table.

Model		q	C	D sep p	CICc	ΔCICc	W
11: OU ~ ANIMALS + ENV + AB, AB ~ ANIMALS ~ ENV	3	63	4.71	0.581	186	0	1.00
14: OU ~ ANIMALS + ENV + FIC, AB ~ ENV + ANIMALS	4	62	20.81	0.008	198	12	0.003
20: OU ~ CERCO + FIC + MAT + P, AB ~ ANIMALS + ENV	5	61	25.03	0.005	198	12.2	0.002
17: OU ~ ENV + FIC + AB, AB ~ ANIMALS + ENV	5	61	25.78	0.004	199	12.9	0.002
13: OU ~ ANIMALS + FIC, AB ~ ANIMALS + ENV	4	62	22.08	0.005	199	13.3	0.001
15: OU ~ ANIMALS + ENV, AB ~ ANIMALS ~ ENV + FIC	2	64	16.43	0.003	202	15.9	< 0.001
19: OU ~ HOM + ENV + FIC, AB ~ ANIMALS + ENV	5	61	31.6	0.001	205	18.8	< 0.001
16: OU ~ FIC ~ ENV, AB ~ ANIMALS + ENV	4	62	31.32	< 0.001	208	22.5	< 0.001
12: OU ~ ANIMALS + FIC + AB, AB ~ ANIMALS ~ ENV	5	61	38.38	< 0.001	211	25.5	< 0.001
18: OU ~ ANIMALS + FIC + P, AB ~ ANIMALS + ENV	5	61	38.38	< 0.001	211	25.5	< 0.001
21: OU ~ FRUG + FIC, AB ~ ANIMALS + ENV + FIC	19	47	513.94	< 0.001	636	449.8	< 0.001
5: OU ~ CERCO, ANIMALS & AB ~ ENV + FIC	22	44	533.48	< 0.001	645	459.5	< 0.001
10: OU ~ FRUG + FIC + ENV, AB ~ ANIMALS + ENV	18	48	530.13	< 0.001	655	469.4	< 0.001
6: OU ~ HOM, ANIMALS & AB ~ ENV + FIC	22	44	623.02	< 0.001	735	549.1	< 0.001
9: OU ~ FIC, ANIMALS & AB ~ ENV	24	42	730.05	< 0.001	836	649.7	< 0.001
1: OU ~ ENV, ANIMALS & AB ~ ENV	23	43	792.4	< 0.001	901	715.2	< 0.001
2: OU ~ ENV, ANIMALS & AB ~ ENV + FIC	22	44	790.99	< 0.001	903	717	< 0.001
4: OU ~ ENV + FIC, ANIMALS & AB ~ ENV	22	44	793.86	< 0.001	906	719.9	< 0.001
7: OU ~ ENV + FIC, AB ~ ANIMALS ~ ENV	22	44	793.86	< 0.001	906	719.9	< 0.001
3: OU ~ CERCO + PTERO + HOM, ANIMALS & AB ~ ENV	24	42	805.36	< 0.001	911	725	< 0.001
8: OU ~ FRUG, ANIMALS & AB ~ ENV	25	41	819.38	< 0.001	922	735.9	< 0.001
22: OU ~ FIC + FRUG, AB ~ ANIMALS ~ ENV + FIC	24	42	823.02	< 0.001	929	742.7	< 0.001

## Data Availability

Data are available from the Dryad Digital Repository: https://doi.org/10.6084/m9.figshare.23110259 (Sundaram et al. 2024).
