# Supplementary material for: Fruit–frugivore dependencies are important in Ebolavirus outbreaks in Sub-Saharan Africa
Source: Ecography. Author manuscript; Available in PMC 2025 Feb 27. (PMC11867621; doi:10.1111/ecog.06950)
Supplement: Supplementary material [file NIHMS2016259-supplement-Supplementary_material.docx]

**Supplemental Information for:**

**Fruit-frugivore dependencies are important in *Ebolavirus* outbreaks**

**in Sub-Saharan Africa**

Mekala Sundaram, Mireya Dorado, Benedicta Akaribo, Antoine Filion, Barbara A Han, Nicole L. Gottdenker, John P Schmidt, John M Drake, Patrick R Stephens

**Table of Contents:**

| ***Ficus* habitat suitability maps** | Page 2 |
| --- | --- |
| **Biological pathways tested with all mammal groups** | Page 3 |
| **Mammals and their diet included in path analyses** | Page 4-9 |
| **Statistics comparing pathways fit to all biological variables** | Page 10 |
| **Biological pathways tested with global *Ficus* suitability** | Page 11 |
| **Biological and environmental pathways tested with global *Ficus***  **suitability** | Page 12-13 |
| **Biological and environmental pathways tested with *Ficus* suitability for**  **African species only** | Page 14-15 |
| **D separation tests for each final path** | Page 16 |
| **Bootstrapped intervals around path coefficients** | Page 17-18 |
| **Statistics for pathways fitted to digitized fig ranges** | Page 19-20 |


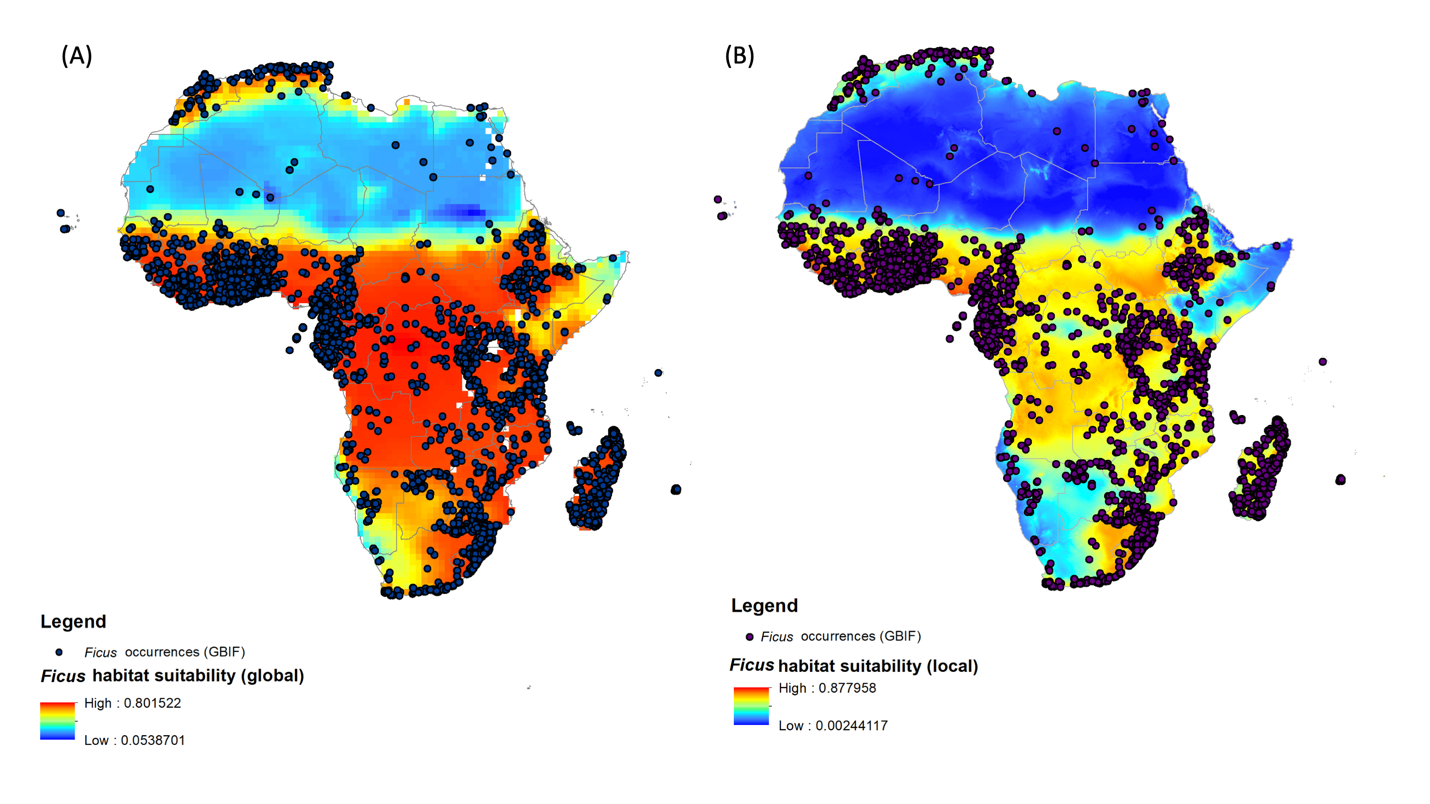


Fig S1. *Ficus* habitat suitability determined from global distribution of *Ficus* (A), *Ficus* habitat suitability determined from African species only (B).


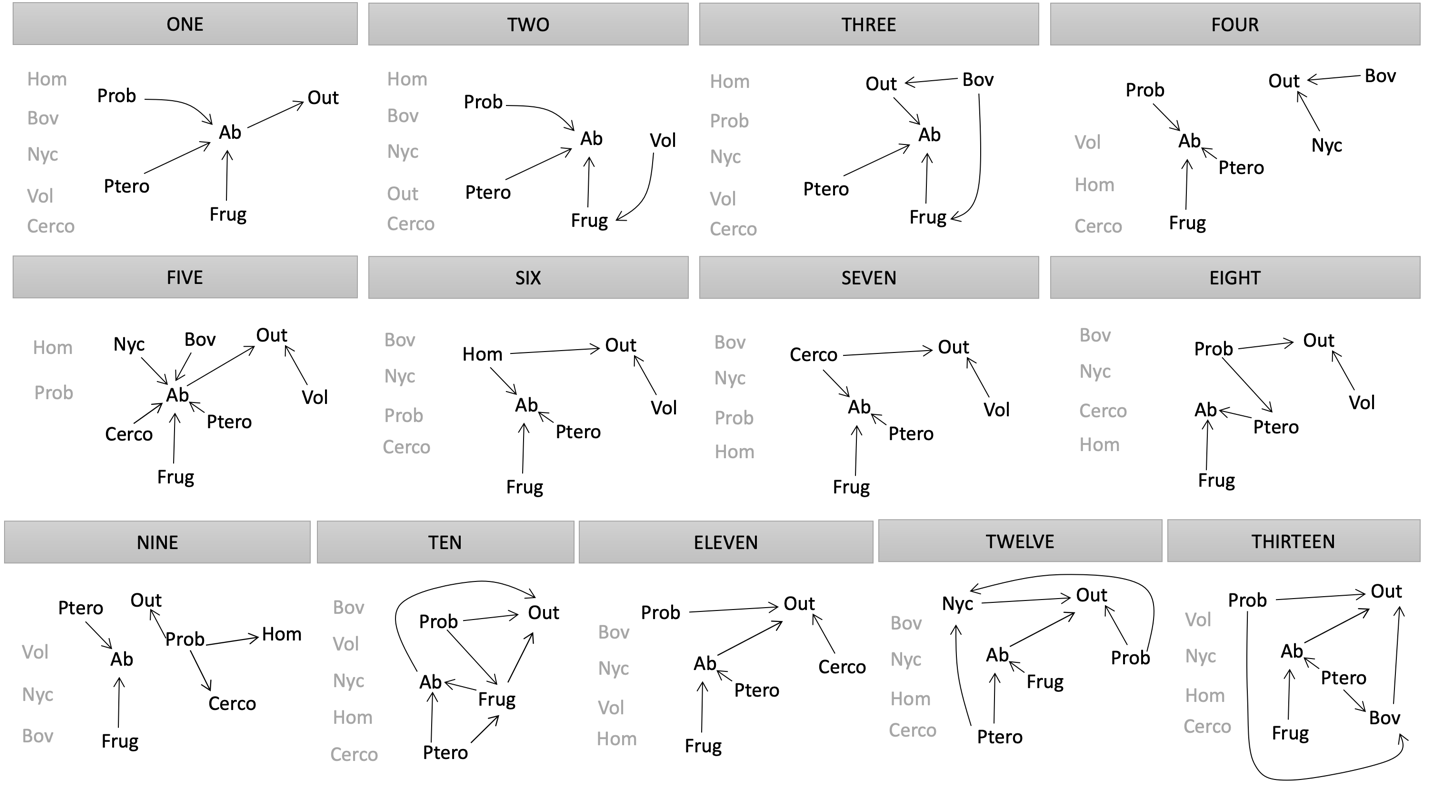


Fig S2. Pathways tested with biological variables: richness of different mammal clades, *Ficus* habitat suitability determined from global distribution of *Ficus*, past infection status of mammal and *Ebolavirus* outbreak occurrence within range of mammal. Variables include outbreak occurrence (out), infection status (ab), *Ficus* habitat suitability (prob), Pteropodidae richness (Ptero), Cercopithecidae richness (Cerco), Frugivore richness (Frug), Hominidae richness (Hom), Nycteridae richness (Nyc), Bovidae richness (Bov), and *Ficus* fruit volume (Vol).

Table S1. The 210 mammals included in the path analyses for whom infection status, range and phylogeny are available. Binomial name on tree from Upham et al. is provided, family of species is provided, percent fruit in diet was obtained from Elton traits database. Frugivores is ‘Yes’ for species with more than 20% of their diet being fruit.

| Tree binomial name | Family | Frugivore | Diet.Fruit |
| --- | --- | --- | --- |
| *Neoromicia_somalicus* | Vespertilionidae | No | 0 |
| *Neoromicia_brunneus* | Vespertilionidae | No | 0 |
| *Allenopithecus_nigroviridis* | Cercopithecidae | Yes | 50 |
| *Anomalurus_derbianus* | Anomaluridae | Yes | 40 |
| *Apodemus_sylvaticus* | Muridae | No | 20 |
| *Atherurus_africanus* | Hystricidae | No | 20 |
| *Canis_aureus* | Canidae | No | 10 |
| *Canis_lupus* | Canidae | No | 0 |
| *Canis_mesomelas* | Canidae | No | 0 |
| *Cardioderma_cor* | Megadermatidae | No | 0 |
| *Casinycteris_argynnis* | Pteropodidae | Yes | 80 |
| *Cephalophus_callipygus* | Bovidae | Yes | 30 |
| *Cercocebus_agilis* | Cercopithecidae | Yes | 70 |
| *Cercocebus_atys* | Cercopithecidae | Yes | 40 |
| *Cercocebus_chrysogaster* | Cercopithecidae | Yes | 70 |
| *Cercocebus_galeritus* | Cercopithecidae | Yes | 70 |
| *Cercocebus_torquatus* | Cercopithecidae | Yes | 70 |
| *Cercopithecus_ascanius* | Cercopithecidae | Yes | 50 |
| *Cercopithecus_campbelli* | Cercopithecidae | Yes | 50 |
| *Cercopithecus_cephus* | Cercopithecidae | Yes | 50 |
| *Cercopithecus_diana* | Cercopithecidae | Yes | 50 |
| *Cercopithecus_erythrotis* | Cercopithecidae | Yes | 50 |
| *Cercopithecus_hamlyni* | Cercopithecidae | Yes | 50 |
| *Cercopithecus_mitis* | Cercopithecidae | Yes | 50 |
| *Cercopithecus_mona* | Cercopithecidae | Yes | 50 |
| *Cercopithecus_neglectus* | Cercopithecidae | Yes | 50 |
| *Cercopithecus_nictitans* | Cercopithecidae | Yes | 50 |
| *Cercopithecus_petaurista* | Cercopithecidae | Yes | 30 |
| *Cercopithecus_pogonias* | Cercopithecidae | Yes | 50 |
| *Cercopithecus_sclateri* | Cercopithecidae | Yes | 50 |
| *Chaerephon_ansorgei* | Molossidae | No | 0 |
| *Chaerephon_chapini* | Molossidae | No | 0 |
| *Chaerephon_major* | Molossidae | No | 0 |
| *Chaerephon_pumilus* | Molossidae | No | 0 |
| *Chlorocebus_aethiops* | Cercopithecidae | Yes | 40 |
| *Chlorocebus_pygerythrus* | Cercopithecidae | Yes | 40 |
| *Chlorocebus_sabaeus* | Cercopithecidae | Yes | 40 |
| *Chlorocebus_tantalus* | Cercopithecidae | Yes | 50 |
| *Coleura_afra* | Emballonuridae | No | 0 |
| *Colobus_angolensis* | Cercopithecidae | Yes | 30 |
| *Colobus_guereza* | Cercopithecidae | Yes | 30 |
| *Colobus_polykomos* | Cercopithecidae | Yes | 30 |
| *Colobus_satanas* | Cercopithecidae | Yes | 30 |
| *Colomys_goslingi* | Muridae | No | 0 |
| *Cricetomys_emini* | Nesomyidae | Yes | 30 |
| *Crocidura_attila* | Soricidae | No | 0 |
| *Crocidura_denti* | Soricidae | No | 0 |
| *Crocidura_dolichura* | Soricidae | No | 0 |
| *Crocidura_hildegardeae* | Soricidae | No | 0 |
| *Crocidura_hirta* | Soricidae | No | 0 |
| *Crocidura_littoralis* | Soricidae | No | 0 |
| *Crocidura_ludia* | Soricidae | No | 0 |
| *Crocidura_montis* | Soricidae | No | 0 |
| *Crocidura_mutesae* | Soricidae | No | 0 |
| *Crocidura_nigrofusca* | Soricidae | No | 0 |
| *Crocidura_olivieri* | Soricidae | No | 0 |
| *Crocidura_parvipes* | Soricidae | No | 0 |
| *Crocidura_poensis* | Soricidae | No | 0 |
| *Crocidura_roosevelti* | Soricidae | No | 0 |
| *Crossarchus_alexandri* | Herpestidae | No | 20 |
| *Dasymys_incomtus* | Muridae | No | 0 |
| *Dasymys_rufulus* | Muridae | No | 0 |
| *Dendrohyrax_dorsalis* | Procaviidae | Yes | 30 |
| *Dendromus_mesomelas* | Nesomyidae | No | 20 |
| *Dendromus_mystacalis* | Nesomyidae | No | 20 |
| *Deomys_ferrugineus* | Muridae | No | 0 |
| *Dephomys_defua* | Muridae | Yes | 90 |
| *Eidolon_dupreanum* | Pteropodidae | Yes | 100 |
| *Eidolon_helvum* | Pteropodidae | Yes | 100 |
| *Epomophorus_gambianus* | Pteropodidae | Yes | 100 |
| *Epomophorus_labiatus* | Pteropodidae | Yes | 100 |
| *Epomophorus_wahlbergi* | Pteropodidae | Yes | 100 |
| *Epomops_buettikoferi* | Pteropodidae | Yes | 100 |
| *Epomops_franqueti* | Pteropodidae | Yes | 100 |
| *Funisciurus_anerythrus* | Sciuridae | Yes | 40 |
| *Funisciurus_congicus* | Sciuridae | Yes | 40 |
| *Funisciurus_pyrropus* | Sciuridae | Yes | 40 |
| *Genetta_servalina* | Viverridae | No | 0 |
| *Gerbilliscus_validus* | Muridae | No | 0 |
| *Glauconycteris_argentata* | Vespertilionidae | No | 0 |
| *Glauconycteris_poensis* | Vespertilionidae | No | 0 |
| *Glauconycteris_variegata* | Vespertilionidae | No | 0 |
| *Gorilla_gorilla* | Hominidae | No | 10 |
| *Grammomys_dolichurus* | Muridae | No | 20 |
| *Graphiurus_lorraineus* | Gliridae | No | 20 |
| *Heimyscus_fumosus* | Muridae | No | 10 |
| *Heliosciurus_gambianus* | Sciuridae | Yes | 30 |
| *Heliosciurus_rufobrachium* | Sciuridae | Yes | 30 |
| *Hipposideros_abae* | Hipposideridae | No | 0 |
| *Hipposideros_beatus* | Hipposideridae | No | 0 |
| *Hipposideros_curtus* | Hipposideridae | No | 0 |
| *Hipposideros_cyclops* | Hipposideridae | No | 0 |
| *Hipposideros_fuliginosus* | Hipposideridae | No | 0 |
| *Hipposideros_jonesi* | Hipposideridae | No | 0 |
| *Hipposideros_ruber* | Hipposideridae | No | 0 |
| *Hybomys_univittatus* | Muridae | Yes | 40 |
| *Hylomyscus_carillus* | Muridae | Yes | 60 |
| *Hylomyscus_parvus* | Muridae | Yes | 60 |
| *Hylomyscus_stella* | Muridae | Yes | 60 |
| *Hypsignathus_monstrosus* | Pteropodidae | Yes | 90 |
| *Hypsugo_musciculus* | Vespertilionidae | No | 0 |
| *Lavia_frons* | Megadermatidae | No | 0 |
| *Lemniscomys_striatus* | Muridae | No | 0 |
| *Lissonycteris_angolensis* | Pteropodidae | Yes | 60 |
| *Lophocebus_albigena* | Cercopithecidae | Yes | 50 |
| *Lophocebus_aterrimus* | Cercopithecidae | Yes | 40 |
| *Lophuromys_nudicaudus* | Muridae | No | 0 |
| *Lophuromys_sikapusi* | Muridae | No | 0 |
| *Malacomys_longipes* | Muridae | No | 20 |
| *Mandrillus_leucophaeus* | Cercopithecidae | Yes | 40 |
| *Mastomys_natalensis* | Muridae | No | 0 |
| *Megaloglossus_woermanni* | Pteropodidae | No | 0 |
| *Micropteropus_pusillus* | Pteropodidae | Yes | 90 |
| *Miniopterus_inflatus* | Vespertilionidae | No | 0 |
| *Miniopterus_minor* | Vespertilionidae | No | 0 |
| *Miniopterus_schreibersii* | Vespertilionidae | No | 0 |
| *Miopithecus_talapoin* | Cercopithecidae | Yes | 50 |
| *Mops_condylurus* | Molossidae | No | 0 |
| *Mops_congicus* | Molossidae | No | 0 |
| *Mops_demonstrator* | Molossidae | No | 0 |
| *Mops_midas* | Molossidae | No | 0 |
| *Mops_nanulus* | Molossidae | No | 0 |
| *Mops_niveiventer* | Molossidae | No | 0 |
| *Mops_thersites* | Molossidae | No | 0 |
| *Mungos_mungo* | Herpestidae | No | 0 |
| *Mus_bufo* | Muridae | No | 0 |
| *Mus_minutoides* | Muridae | No | 0 |
| *Mus_musculoides* | Muridae | No | 0 |
| *Mus_musculus* | Muridae | No | 0 |
| *Mus_triton* | Muridae | No | 0 |
| *Myonycteris_torquata* | Pteropodidae | Yes | 80 |
| *Myopterus_whitleyi* | Molossidae | No | 0 |
| *Myotis_bocagii* | Vespertilionidae | No | 0 |
| *Myotis_tricolor* | Vespertilionidae | No | 0 |
| *Myotis_welwitschii* | Vespertilionidae | No | 0 |
| *Nandinia_binotata* | Nandiniidae | Yes | 80 |
| *Nanonycteris_veldkampii* | Pteropodidae | No | 0 |
| *Neoromicia_rendalli* | Vespertilionidae | No | 0 |
| *Neoromicia_tenuipinnis* | Vespertilionidae | No | 0 |
| *Neotragus_pygmaeus* | Bovidae | No | 20 |
| *Nycteris_grandis* | Nycteridae | No | 0 |
| *Nycteris_hispida* | Nycteridae | No | 0 |
| *Nycteris_thebaica* | Nycteridae | No | 0 |
| *Oenomys_hypoxanthus* | Muridae | No | 0 |
| *Otomops_martiensseni* | Molossidae | No | 0 |
| *Pan_paniscus* | Hominidae | Yes | 60 |
| *Pan_troglodytes* | Hominidae | Yes | 60 |
| *Papio_anubis* | Cercopithecidae | No | 20 |
| *Papio_cynocephalus* | Cercopithecidae | No | 20 |
| *Papio_hamadryas* | Cercopithecidae | No | 0 |
| *Paracrocidura_schoutedeni* | Soricidae | No | 0 |
| *Paraxerus_alexandri* | Sciuridae | Yes | 40 |
| *Pelomys_campanae* | Muridae | No | 0 |
| *Pelomys_minor* | Muridae | No | 0 |
| *Perodicticus_potto* | Lorisidae | Yes | 80 |
| *Petrodromus_tetradactylus* | Macroscelididae | No | 0 |
| *Philantomba_monticola* | Bovidae | Yes | 70 |
| *Piliocolobus_badius* | Cercopithecidae | No | 20 |
| *Piliocolobus_pennantii* | Cercopithecidae | No | 20 |
| *Pipistrellus_hesperidus* | Vespertilionidae | No | 0 |
| *Pipistrellus_inexspectatus* | Vespertilionidae | No | 0 |
| *Pipistrellus_kuhlii* | Vespertilionidae | No | 0 |
| *Pipistrellus_nanulus* | Vespertilionidae | No | 0 |
| *Potamochoerus_porcus* | Suidae | Yes | 40 |
| *Praomys_derooi* | Muridae | No | 0 |
| *Praomys_jacksoni* | Muridae | Yes | 30 |
| *Praomys_lukolelae* | Muridae | No | 20 |
| *Praomys_misonnei* | Muridae | Yes | 30 |
| *Praomys_tullbergi* | Muridae | Yes | 30 |
| *Procolobus_verus* | Cercopithecidae | No | 10 |
| *Protoxerus_stangeri* | Sciuridae | Yes | 30 |
| *Pteropus_rufus* | Pteropodidae | Yes | 100 |
| *Rattus_norvegicus* | Muridae | No | 20 |
| *Rhinolophus_alcyone* | Rhinolophidae | No | 0 |
| *Rhinolophus_blasii* | Rhinolophidae | No | 0 |
| *Rhinolophus_clivosus* | Rhinolophidae | No | 0 |
| *Rhinolophus_darlingi* | Rhinolophidae | No | 0 |
| *Rhinolophus_euryale* | Rhinolophidae | No | 0 |
| *Rhinolophus_ferrumequinum* | Rhinolophidae | No | 0 |
| *Rhinolophus_fumigatus* | Rhinolophidae | No | 0 |
| *Rhinolophus_landeri* | Rhinolophidae | No | 0 |
| *Rhinolophus_mehelyi* | Rhinolophidae | No | 0 |
| *Rhinopoma_microphyllum* | Rhinopomatidae | No | 0 |
| *Rhynchocyon_cirnei* | Macroscelididae | No | 0 |
| *Rousettus_aegyptiacus* | Pteropodidae | Yes | 60 |
| *Rousettus_lanosus* | Pteropodidae | Yes | 60 |
| *Rousettus_madagascariensis* | Pteropodidae | Yes | 60 |
| *Ruwenzorisorex_suncoides* | Soricidae | No | 0 |
| *Saccolaimus_peli* | Emballonuridae | No | 0 |
| *Scotoecus_hirundo* | Vespertilionidae | No | 0 |
| *Scotonycteris_zenkeri* | Pteropodidae | Yes | 100 |
| *Scotophilus_dinganii* | Vespertilionidae | No | 0 |
| *Scotophilus_leucogaster* | Vespertilionidae | No | 0 |
| *Scotophilus_nigrita* | Vespertilionidae | No | 0 |
| *Scotophilus_nux* | Vespertilionidae | No | 0 |
| *Scotophilus_viridis* | Vespertilionidae | No | 0 |
| *Scutisorex_somereni* | Soricidae | No | 0 |
| *Steatomys_pratensis* | Nesomyidae | No | 0 |
| *Stochomys_longicaudatus* | Muridae | Yes | 80 |
| *Suncus_murinus* | Soricidae | No | 0 |
| *Suncus_remyi* | Soricidae | No | 0 |
| *Sus_scrofa* | Suidae | No | 0 |
| *Sylvicapra_grimmia* | Bovidae | Yes | 20 |
| *Sylvisorex_ollula* | Soricidae | No | 0 |
| *Taphozous_mauritianus* | Emballonuridae | No | 0 |
| *Taphozous_perforatus* | Emballonuridae | No | 0 |
| *Thryonomys_swinderianus* | Thryonomyidae | No | 10 |
| *Tragelaphus_scriptus* | Bovidae | No | 0 |
| *Triaenops_persicus* | Hipposideridae | No | 0 |
| *Uranomys_ruddi* | Muridae | No | 0 |

Table S2. Phylogenetic pathways tested for biological variables diagrammed in Fig S1. The table summarizes the model number (model), the number of independence tests performed (k), the number of parameters in the model (q), C-statistic (C), p value associated with d separation tests (D sep p), C-statistic corrected for small sample sizes (CICc), difference between model CICc value and the smallest CICc value in set (ΔCICc) and weight of model relative to other models in set (w).

| model | k | q | C | D sep  p | CICc | ΔCICc | w |
| --- | --- | --- | --- | --- | --- | --- | --- |
| ten | 38 | 17 | 1489 | 0 | 1527 | 0 | 1.00 |
| thirteen | 38 | 17 | 1526 | 0 | 1564 | 37 | <0.0001 |
| twelve | 38 | 17 | 1550 | 0 | 1588 | 61 | <0.0001 |
| eight | 40 | 15 | 1600 | 0 | 1633 | 106 | <0.0001 |
| nine | 40 | 15 | 1653 | 0 | 1685 | 159 | <0.0001 |
| three | 40 | 15 | 1707 | 0 | 1740 | 213 | <0.0001 |
| four | 40 | 15 | 1716 | 0 | 1748 | 221 | <0.0001 |
| two | 41 | 14 | 1721 | 0 | 1752 | 225 | <0.0001 |
| eleven | 40 | 15 | 1729 | 0 | 1761 | 235 | <0.0001 |
| seven | 40 | 15 | 1732 | 0 | 1765 | 238 | <0.0001 |
| six | 40 | 15 | 1746 | 0 | 1778 | 252 | <0.0001 |
| five | 38 | 17 | 1789 | 0 | 1826 | 300 | <0.0001 |
| one | 41 | 14 | 1890 | 0 | 1920 | 394 | <0.0001 |


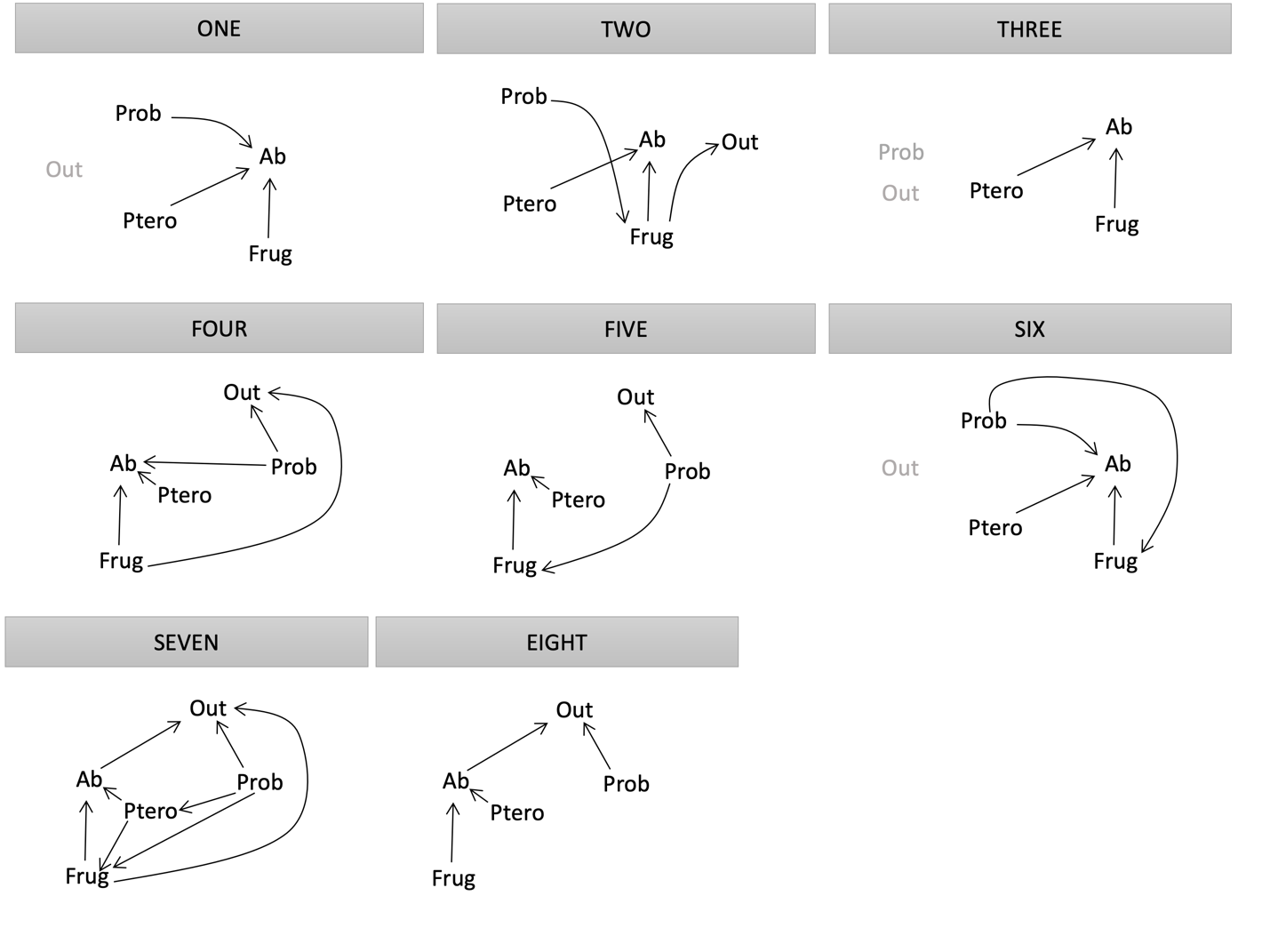


Fig S3. Pathways tested with biological variables: richness of Pteropodidae bats, frugivorous mammals, *Ficus* habitat suitability determined from global distribution of *Ficus*, past infection status of mammal and *Ebolavirus* outbreak occurrence within range of mammal. Variables include outbreak occurrence (out), infection status (ab), *Ficus* habitat suitability (prob), Pteropodidae richness (Ptero), and Frugivore richness (Frug).


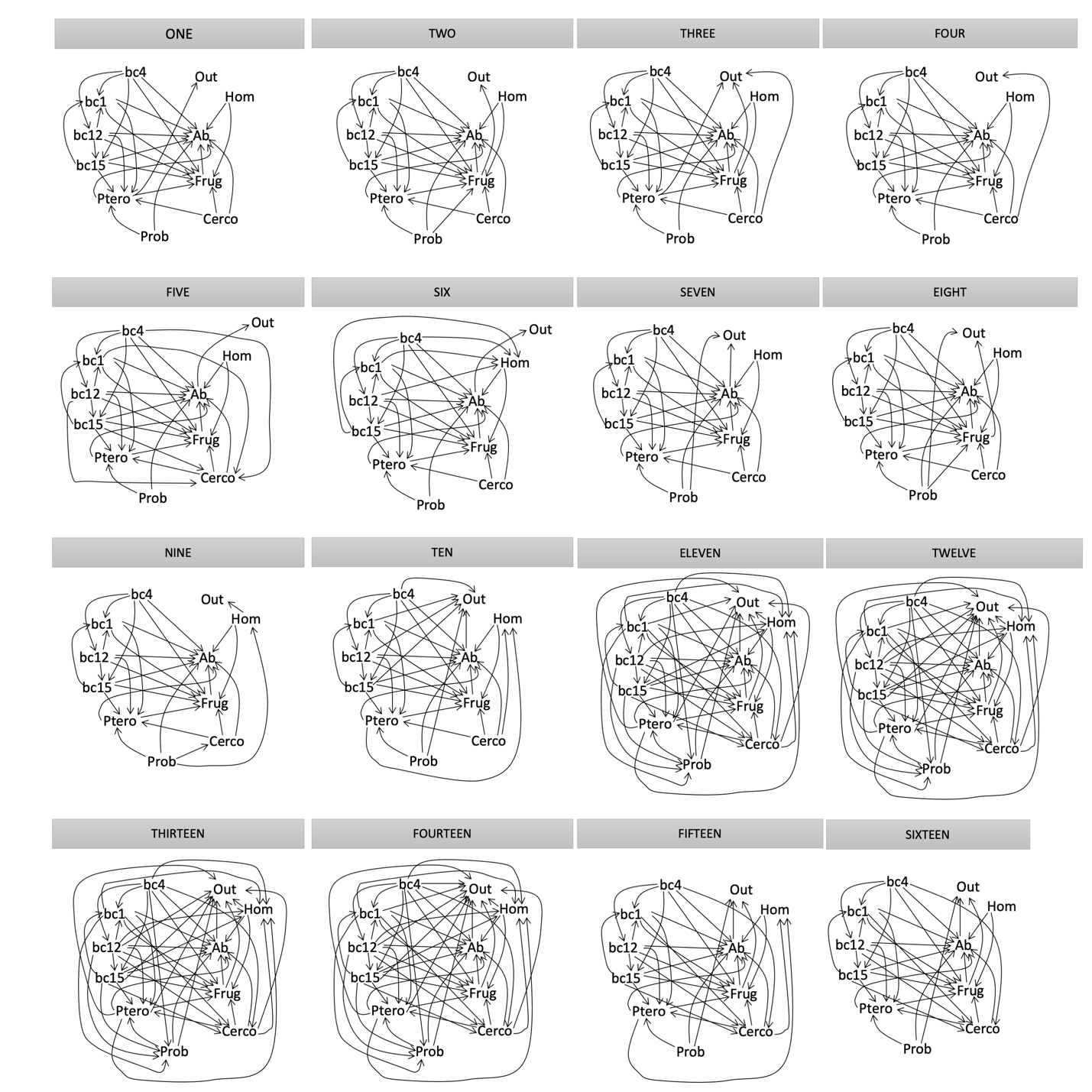


Fig S4. Pathways tested with both biological and environmental variables. Here *Ficus* habitat suitability was estimated from global distribution of all *Ficus* species. Variables include outbreak occurrence (out), infection status (ab), *Ficus* habitat suitability (prob), Pteropodidae richness (Ptero), Cercopithecidae richness (Cerco), Frugivore richness (Frug), Hominidae richness (Hom), mean annual temperature (bc1), mean annual precipitation (bc12), temperature seasonality (bc4), and precipitation seasonality (bc15).


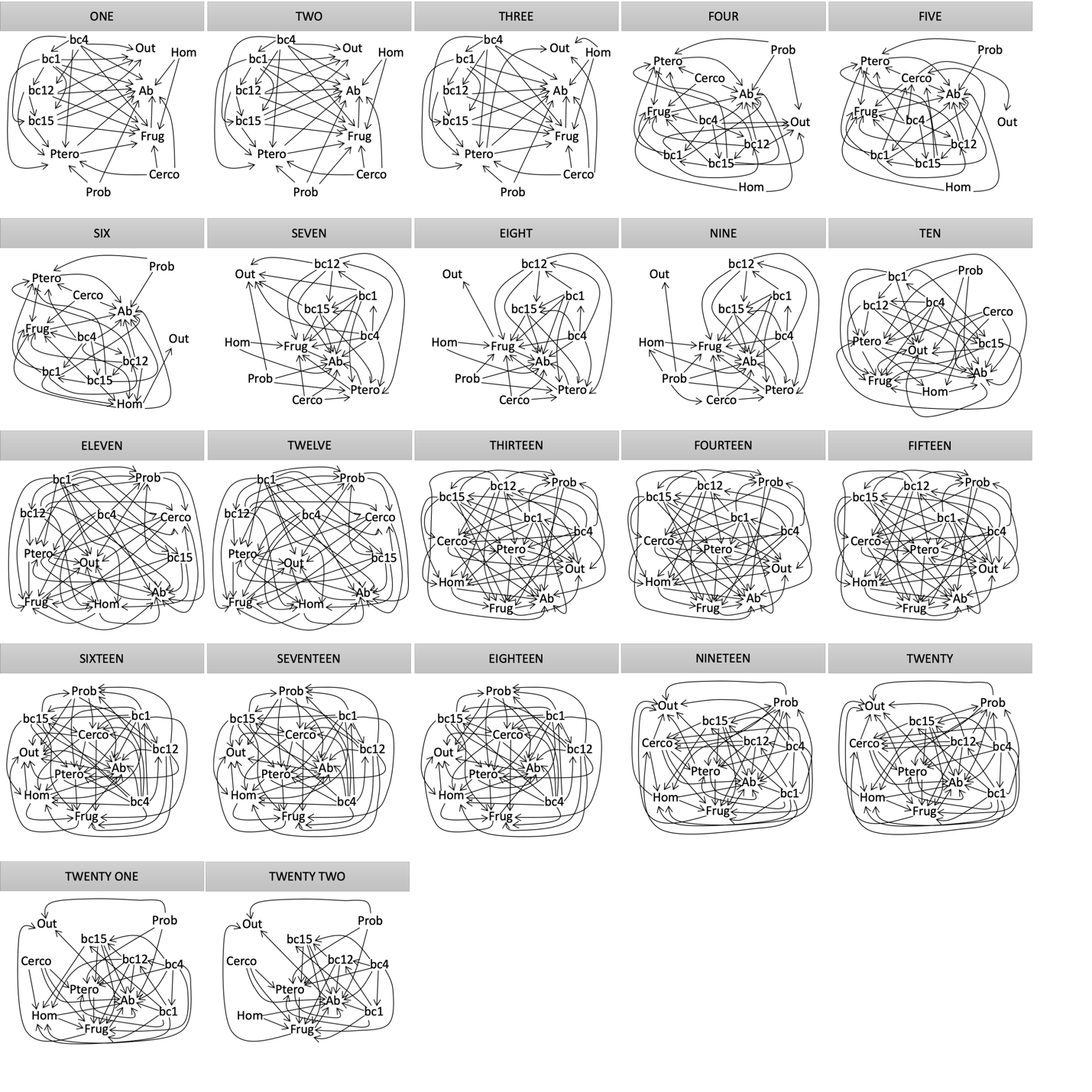


Fig S5. Pathways tested with both biological and environmental variables. Here *Ficus* habitat suitability was estimated from distribution of African *Ficus* species only. Variables include outbreak occurrence (out), infection status (ab), *Ficus* habitat suitability (prob), Pteropodidae richness (Ptero), Cercopithecidae richness (Cerco), Frugivore richness (Frug), Hominidae richness (Hom), mean annual temperature (bc1), mean annual precipitation (bc12), temperature seasonality (bc4), and precipitation seasonality (bc15).

Table S3. Summary of d-separation test results for all best pathways presented in Tables 1, 2 and 3 of main paper.

| D-sep for best model in Table-1 of main text including only biological variables: *Ficus* habitat suitability estimated from global *Ficus* points, Pteropodidae richness, frugivore richness predicting antibodies and outbreak occurrence | | | |
| --- | --- | --- | --- |
| D sep model | D sep p | model |  |
| ab~ptero+frug+prob | 0.587 | phyloglm |  |
| out~prob+frug+ab+ptero | 0.351 | phyloglm |  |
|  |  |  |  |
| D-sep for best model in Table-2 of main text with both biological and environmental variables: *Ficus* habitat suitability estimated from global *Ficus* points, mammal group richness, environmental variables predicting antibody test results and outbreak occurrence | | | |
| D sep model | D sep p | phylo_par | model |
| out ~ bc12 + bc15 + cerco + prob + ptero + hom + frug + ab + bc4 | 0.05 | NA | phyloglm |
| out ~ bc4 + bc12 + bc15 + cerco + prob + ptero + hom + frug + ab + bc1 | 0.978 | NA | phyloglm |
| cerco ~ bc4 + bc12 + bc15 + bc1 + prob | 0.186 | 0.0454 | phylolm |
| hom ~ bc4 + bc12 + bc15 + bc1 + cerco + ptero + prob | 0.237 | <0.0001 | phylolm |
| frug ~ bc4 + bc12 + bc15 + bc1 + cerco + ptero + hom + prob | 0.135 | 0.0568 | phylolm |
|  |  |  |  |
| D-sep for best model in Table-3 of main text with both biological and environmental variables: *Ficus* habitat suitability estimated with African points only, mammal group richness, environmental variables predicting antibody test results and outbreak occurrence | | | |
| D sep model | D sep p | phylo_par | model |
| out ~ bc1 + bc12 + prob + cerco + ptero + hom + frug + ab + bc4 | 0.191 | NA | phyloglm |
| out ~ bc4 + bc1 + bc12 + prob + cerco + ptero + hom + frug + ab + bc15 | 1 | NA | phyloglm |
| frug ~ bc4 + bc1 + bc12 + bc15 + cerco + ptero + hom + prob | 0.497 | <0.001 | phylolm |

Table S4. Bootstrapped confidence intervals for path coefficients generated from 199 repeats. For each row variable predicting a column variable, the upper row provides the lower confidence limit and the second row provides the upper confidence limit. For example, the confidence interval around bc4 predicting bc1 is -0.7 to -0.48 for both pathways presented. Models used to generate the confidence intervals are best pathways (see main text for more details) first for *Ficus* habitat suitability generated with global *Ficus* points and next with *Ficus* points for Africa only. Variables are bc4 (temperature seasonality), bc1 (mean annual temperature), bc12 (mean annual precipitation), bc15 (precipitation seasonality), prob (*Ficus* habitat suitability), cerco (Cercopithecidae richness), Ptero (Pteropodidae richness), Hom (Hominidae richness), frug (Frugivore richness), ab (positive past infection determined from antibody and PCR tests), and out (ranges that overlap *Ebolavirus* outbreaks).

| Best pathway in Table-2 of main text with both biological and environmental variables: *Ficus* habitat suitability estimated from global *Ficus* points, mammal group richness, environmental variables predicting antibody test results and outbreak occurrence | | | | | | | | | | | |
| --- | --- | --- | --- | --- | --- | --- | --- | --- | --- | --- | --- |
|  | bc4 | bc12 | bc15 | bc1 | cerco | prob | ptero | hom | frug | ab | out |
| bc4 |  | -0.84 | -0.28 | -0.62 | -0.41 | -0.87 | -0.33 | 0.19 | -0.06 | -0.28 | 0.00 |
|  |  | -0.66 | 0.04 | -0.33 | -0.28 | -0.62 | -0.05 | 0.41 | 0.05 | 0.99 | 0.00 |
| bc12 |  |  | -0.71 | 0.23 | 0.21 | 0.19 | 0.41 | 0.02 | 0.07 | -0.87 | -2.17 |
|  |  |  | -0.42 | 0.51 | 0.34 | 0.47 | 0.66 | 0.31 | 0.20 | 0.49 | -0.32 |
| bc15 |  |  |  | 0.27 | -0.53 | 0.18 | 0.12 | -0.14 | 0.03 | -0.31 | 0.19 |
|  |  |  |  | 0.52 | -0.43 | 0.36 | 0.35 | 0.13 | 0.14 | 0.81 | 1.22 |
| bc1 |  |  |  |  | 0.07 | -0.46 | -0.20 | -0.14 | -0.20 | -0.83 | 0.00 |
|  |  |  |  |  | 0.17 | -0.24 | -0.02 | 0.02 | -0.12 | 0.43 | 0.00 |
| cerco |  |  |  |  |  | 0.00 | 0.30 | 0.82 | 0.52 | -0.08 | 0.98 |
|  |  |  |  |  |  | 0.00 | 0.67 | 1.35 | 0.74 | 2.06 | 3.19 |
| prob |  |  |  |  |  |  | -0.17 | 0.00 | 0.00 | 0.01 | 0.16 |
|  |  |  |  |  |  |  | 0.01 | 1.35 | 0.00 | 1.09 | 1.18 |
| ptero |  |  |  |  |  |  |  | -0.19 | 0.27 | -0.44 | -0.97 |
|  |  |  |  |  |  |  |  | 0.09 | 0.37 | 0.93 | 0.68 |
| hom |  |  |  |  |  |  |  |  | 0.02 | -0.23 | -0.84 |
|  |  |  |  |  |  |  |  |  | 0.14 | 1.06 | 1.17 |
| frug |  |  |  |  |  |  |  |  |  | -3.08 | -0.22 |
|  |  |  |  |  |  |  |  |  |  | -0.05 | 1.94 |
| ab |  |  |  |  |  |  |  |  |  |  | -0.50 |
|  |  |  |  |  |  |  |  |  |  |  | 0.95 |
|  |  |  |  |  |  |  |  |  |  |  |  |
| Best pathway in Table-3 of main text with both biological and environmental variables: *Ficus* habitat suitability based on African points only, mammal group richness, environmental variables predicting antibody test results and outbreak occurrence | | | | | | | | | | | |
|  | bc4 | bc1 | bc12 | bc15 | prob | cerco | ptero | hom | frug | ab | out |
| bc4 |  | -0.73 | -0.80 | -0.04 | -0.68 | -0.52 | 0.03 | 0.07 | -0.06 | 0.11 | 0.00 |
|  |  | -0.52 | -0.61 | 0.27 | -0.46 | -0.36 | 0.27 | 0.33 | 0.06 | 2.13 | 0.00 |
| bc1 |  |  | -0.01 | 0.38 | -0.31 | 0.06 | -0.03 | -0.16 | -0.20 | -1.72 | 0.44 |
|  |  |  | 0.15 | 0.65 | -0.13 | 0.17 | 0.13 | -0.01 | -0.13 | -0.47 | 1.20 |
| bc12 |  |  |  | -0.79 | 0.41 | 0.22 | 0.03 | 0.06 | 0.06 | -0.08 | -4.04 |
|  |  |  |  | -0.52 | 0.64 | 0.38 | 0.29 | 0.31 | 0.18 | 1.49 | -2.26 |
| bc15 |  |  |  |  | 0.27 | -0.50 | 0.11 | -0.08 | 0.05 | 0.22 | 0.00 |
|  |  |  |  |  | 0.44 | -0.39 | 0.28 | 0.15 | 0.13 | 1.86 | 0.00 |
| prob |  |  |  |  |  | -0.18 | 0.21 | -0.31 | 0.00 | 0.00 | 0.46 |
|  |  |  |  |  |  | -0.02 | 0.42 | -0.09 | 0.00 | 1.60 | 2.15 |
| cerco |  |  |  |  |  |  | 0.63 | 0.78 | 0.53 | 2.18 | 1.65 |
|  |  |  |  |  |  |  | 0.95 | 1.16 | 0.74 | 4.99 | 3.55 |
| ptero |  |  |  |  |  |  |  | -0.01 | 0.28 | 0.60 | -1.85 |
|  |  |  |  |  |  |  |  | 0.25 | 0.40 | 2.92 | -0.03 |
| hom |  |  |  |  |  |  |  |  | 0.03 | 0.59 | -1.18 |
|  |  |  |  |  |  |  |  |  | 0.15 | 2.36 | 0.49 |
| frug |  |  |  |  |  |  |  |  |  | -6.97 | 1.09 |
|  |  |  |  |  |  |  |  |  |  | -3.31 | 2.94 |
| ab |  |  |  |  |  |  |  |  |  |  | -0.40 |
|  |  |  |  |  |  |  |  |  |  |  | 1.38 |

Table S5. Sensitivity of path analyses to *Ficus* richness defined by georeferencing and digitizing fig range images from figweb.org. Pathways in Fig S3 and Fig S4 tested against data. The table summarizes the model number (model), the number of independence tests performed (k), the number of parameters in the model (q), C-statistic (C), p value associated with d separation tests (D sep p), C-statistic corrected for small sample sizes (CICc), difference between model CICc value and the smallest CICc value in set (ΔCICc) and weight of model relative to other models in set (w).

| Pathways with only biological variables represented in Fig S3 | | | | | | | |
| --- | --- | --- | --- | --- | --- | --- | --- |
| model | k | q | C | p | CICc | delta_CICc | w |
| seven | 2 | 13 | 4.31 | 0.366 | 32.2 | 0 | 1.00 |
| two | 6 | 9 | 142.51 | 0 | 161.4 | 129 | <0.001 |
| five | 6 | 9 | 143.6 | 0 | 162.5 | 130 | <0.001 |
| six | 6 | 9 | 216.38 | 0 | 235.3 | 203 | <0.001 |
| four | 5 | 10 | 216.79 | 0 | 237.9 | 206 | <0.001 |
| eight | 6 | 9 | 224.28 | 0 | 243.2 | 211 | <0.001 |
| one | 7 | 8 | 333.31 | 0 | 350 | 318 | <0.001 |
| three | 8 | 7 | 335.99 | 0 | 350.5 | 318 | <0.001 |
| Pathways with both biological and environmental variables represented in Fig S4 | | | | | | | |
| model | k | q | C | p | CICc | delta_CICc | w |
| thirteen | 5 | 61 | 69 | <0.001 | 242 | 0 | 0.869 |
| twelve | 4 | 62 | 69 | <0.001 | 246 | 4.03 | 0.116 |
| fourteen | 3 | 63 | 69 | <0.001 | 250 | 8.12 | 0.015 |
| eleven | 6 | 60 | 87.5 | <0.001 | 257 | 14.58 | 0.001 |
| ten | 18 | 48 | 545.9 | 0 | 671 | 429.02 | <0.001 |
| nine | 25 | 41 | 585.6 | 0 | 688 | 446.04 | <0.001 |
| fifteen | 22 | 44 | 594.5 | 0 | 707 | 464.43 | <0.001 |
| five | 23 | 43 | 707.7 | 0 | 817 | 574.43 | <0.001 |
| six | 23 | 43 | 755.6 | 0 | 864 | 622.32 | <0.001 |
| sixteen | 24 | 42 | 913.9 | 0 | 1020 | 777.47 | <0.001 |
| two | 26 | 40 | 1015.3 | 0 | 1115 | 872.59 | <0.001 |
| eight | 26 | 40 | 1015.3 | 0 | 1115 | 872.59 | <0.001 |
| seven | 27 | 39 | 1019.9 | 0 | 1116 | 874.2 | <0.001 |
| three | 27 | 39 | 1019.9 | 0 | 1116 | 874.2 | <0.001 |
| one | 27 | 39 | 1019.9 | 0 | 1116 | 874.2 | <0.001 |
| four | 27 | 39 | 1019.9 | 0 | 1116 | 874.2 | <0.001 |
